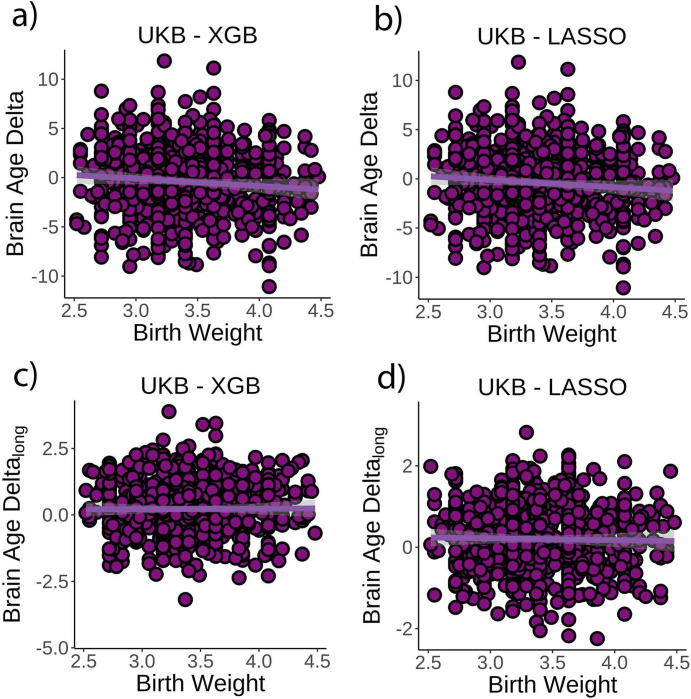# Correction: Individual variations in ‘Brain Age’ relate to early-life factors more than to longitudinal brain change

**DOI:** 10.7554/eLife.79475

**Published:** 2022-04-26

**Authors:** Didac Vidal-Pineiro, Yunpeng Wang, Stine K Krogsrud, Inge K Amlien, William FC Baaré, David Bartres-Faz, Lars Bertram, Andreas M Brandmaier, Christian A Drevon, Sandra Düzel, Klaus Ebmeier, Richard N Henson, Carme Junqué, Rogier Andrew Kievit, Simone Kühn, Esten Leonardsen, Ulman Lindenberger, Kathrine S Madsen, Fredrik Magnussen, Athanasia Monika Mowinckel, Lars Nyberg, James M Roe, Barbara Segura, Stephen M Smith, Øystein Sørensen, Sana Suri, Rene Westerhausen, Andrew Zalesky, Enikő Zsoldos, Kristine Beate Walhovd, Anders Fjell

**Keywords:** Human

Vidal-Pineiro D, Wang Y, Krogsrud SK, Amlien IK, Baaré WFC, Bartres-Faz D, Bertram L, Brandmaier AM, Drevon CA, Düzel S, Ebmeier K, Henson RN, Junqué C, Kievit RA, Kühn S, Leonardsen E, Lindenberger E, Madsen KS, Magnussen F, Mowinckel AM, Nyberg L, Roe JM, Segura B, Smith SM, Sørensen Ø, Suri S, Westerhausen R, Zalesky A, Zsoldos E, Walhovd KB, Fjell A. 2021. Individual variations in ‘brain age’ relate to early-life factors more than to longitudinal brain change. *eLife* **10**:e69995. doi: 10.7554/eLife.69995.

Published 10 November 2021

We discovered that Figure 3 and Figure 4 were “switched”, i.e., Figure 3 was represented in Figure 4 and vice versa. Also, Figure 4, panel (d) represented the information on Figure 3, panel (d). The error was due to an incorrect Figure-name assignment when saved. The error referred only to the visual representation. Both, the caption to the Figures and the statistics are correct. The article has been corrected accordingly. The updated figures are shown below. Finally, the funding source for sigma2 is nn9769k, instead of NN9767k.

The corrected Figure 3:

**Figure 1:**
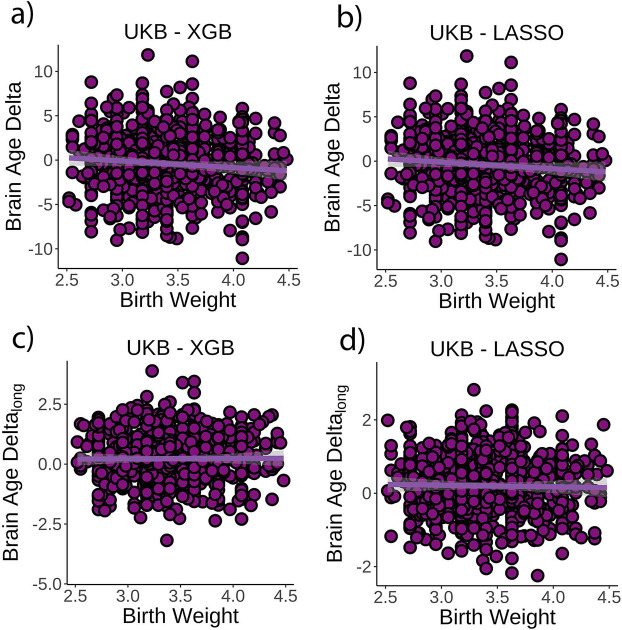


The originally published Figure 3 is shown here for reference:

**Figure 2:**
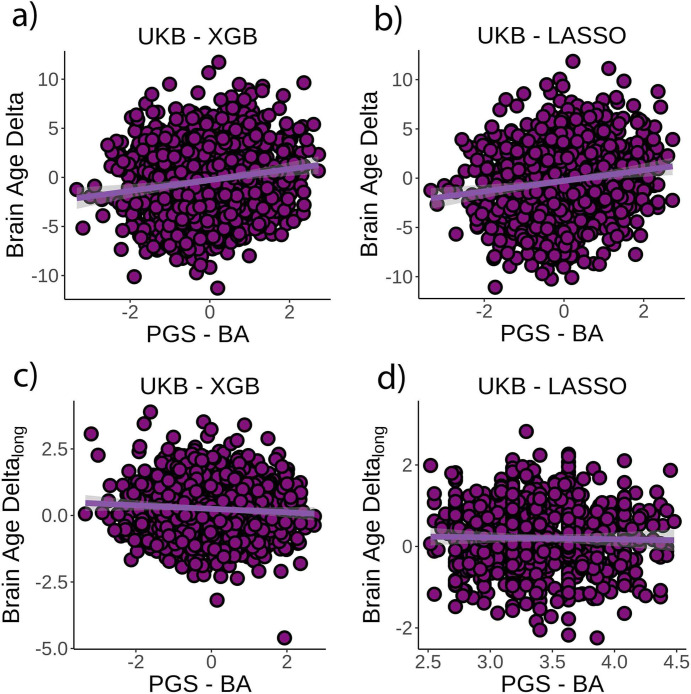


The corrected Figure 4:

**Figure 3:**
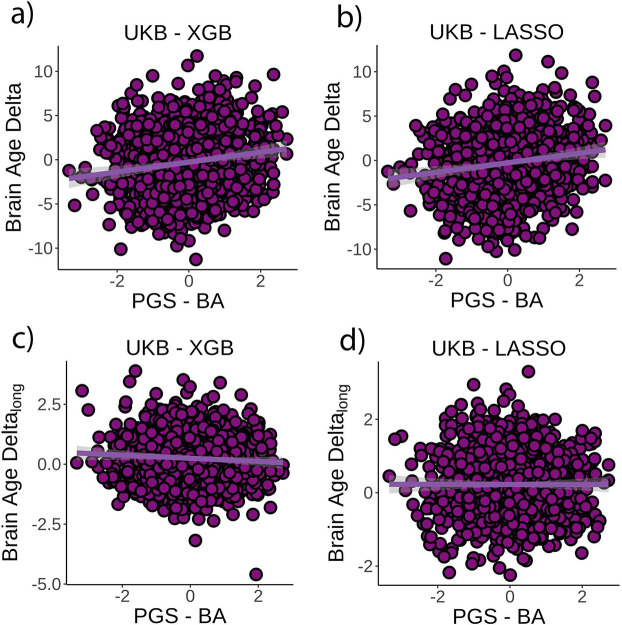


The originally published Figure 4 is shown here for reference:

**Figure 4:**