# Supplementary material for: Individual variations in ‘brain age’ relate to early-life factors more than to longitudinal brain change
Source: eLife. 2021 Nov 10;10:e69995. doi: 10.7554/eLife.69995 (PMC8580481; doi:10.7554/eLife.69995)
Supplement: Supplementary file 1. — List of cortical features included in the brain age model and age variance explained in the UK Biobank and the Lifebrain training datasets. Vol = volume; GWC = gray-white matter contrast; Cth = cortical thickness. [file elife-69995-supp1.docx]

|  | **GWC** | **Vol** | **Area** | **Cth** | **GWC** | **Vol** | **Area** | **Cth** | **GWC** | **Vol** | **Area** | **Cth** | **GWC** | **Vol** | **Area** | **Cth** |
| --- | --- | --- | --- | --- | --- | --- | --- | --- | --- | --- | --- | --- | --- | --- | --- | --- |
|  | **UB Biobank** | | | | | | | | **Lifebrain** | | | | | | | |
|  | **Left hemisphere** | | | | **Right hemisphere** | | | | **Left hemisphere** | | | | **Right hemisphere** | | | |
| **Total surface** | -- | -- | .00 | .10 | -- | .. | .00 | .10 |  |  | .17 | .60 |  |  | .16 | .58 |
| **Cingulate, caudal ant** | .06 | .01 | .00 | .01 | .07 | .01 | .00 | .00 | .38 | .09 | .02 | .09 | .40 | .13 | .05 | .11 |
| **Cingulate, rostral ant** | .20 | .01 | .00 | .02 | .15 | .01 | .00 | .00 | .52 | .16 | .03 | .26 | .44 | .14 | .06 | .12 |
| **Cingulate, posterior** | .13 | .01 | .00 | .02 | .16 | .02 | .01 | .03 | .48 | .23 | .09 | .30 | .52 | .22 | .10 | .27 |
| **Cingulate, isthmus** | .06 | .00 | .00 | .03 | .04 | .00 | .00 | .02 | .50 | .14 | .01 | .29 | .48 | .14 | .02 | .25 |
| **Insula** | .09 | .00 | .00 | .02 | .10 | .00 | .00 | .02 | .39 | .17 | .00 | .42 | .40 | .15 | .00 | .44 |
| **Frontal, superior** | .26 | .05 | .00 | .14 | .27 | .04 | .00 | .13 | .65 | .40 | .11 | .53 | .66 | .37 | .12 | .45 |
| **Frontal, caudal middle** | .18 | .03 | .00 | .08 | .20 | .02 | .00 | .07 | .60 | .23 | .06 | .42 | .61 | .20 | .06 | .39 |
| **Frontal, rostral middle** | .22 | .04 | .01 | .11 | .23 | .04 | .01 | .08 | .61 | .45 | .16 | .43 | .60 | .35 | .14 | .34 |
| **Frontal, pars opercularis** | .20 | .03 | .01 | .07 | .22 | .03 | .00 | .06 | .61 | .30 | .10 | .51 | .63 | .30 | .09 | .48 |
| **Frontal, pars triangularis** | .18 | .04 | .01 | .07 | .21 | .04 | .01 | .08 | .62 | .34 | .12 | .47 | .63 | .31 | .10 | .46 |
| **Frontal, pars orbitalis** | .16 | .04 | .01 | .04 | .19 | .05 | .01 | .04 | .56 | .37 | .16 | .26 | .58 | .38 | .16 | .26 |
| **Frontal, lateral orbital** | .18 | .03 | .02 | .02 | .16 | .03 | .01 | .01 | .54 | .39 | .18 | .32 | .49 | .31 | .11 | .19 |
| **Frontal, medial orbital** | .20 | .02 | .00 | .02 | .22 | .03 | .00 | .01 | .53 | .19 | .03 | .27 | .53 | .24 | .08 | .16 |
| **Frontal, pole** | .19 | .02 | .01 | .03 | .17 | .01 | .01 | .02 | .53 | .27 | .14 | .12 | .47 | .21 | .09 | .07 |
| **Frontal, precentral gyrus** | .13 | .04 | .00 | .09 | .15 | .04 | .00 | .10 | .61 | .25 | .01 | .45 | .63 | .26 | .01 | .45 |
| **Parietal, postcentral gyrus** | .09 | .02 | .00 | .06 | .09 | .02 | .00 | .06 | .53 | .20 | .03 | .37 | .52 | .19 | .02 | .36 |
| **Parietal, paracentral gyrus** | .12 | .03 | .00 | .07 | .13 | .03 | .00 | .07 | .54 | .17 | .02 | .30 | .57 | .15 | .01 | .26 |
| **Parietal, superior** | .15 | .03 | .00 | .07 | .13 | .04 | .00 | .08 | .53 | .26 | .10 | .30 | .53 | .29 | .11 | .32 |
| **Parietal, inferior** | .16 | .03 | .00 | .08 | .14 | .04 | .00 | .11 | .53 | .29 | .12 | .40 | .54 | .32 | .11 | .46 |
| **Parietal, supramarginal** | .16 | .02 | .00 | .09 | .18 | .02 | .00 | .09 | .55 | .27 | .08 | .53 | .57 | .28 | .05 | .54 |
| **Parietal, precuneus** | .20 | .05 | .00 | .09 | .18 | .03 | .00 | .08 | .57 | .31 | .11 | .47 | .56 | .28 | .10 | .43 |
| **Temporal, parahippocampal** | .06 | .03 | .02 | .02 | .06 | .02 | .01 | .02 | .24 | .15 | .12 | .06 | .32 | .16 | 8.0 | .10 |
| **Temporal, entorhinal** | .09 | .00 | .00 | .04 | .09 | .00 | .00 | .03 | .25 | .02 | .01 | .07 | .25 | .01 | .00 | .07 |
| **Temporal, pole** | .10 | -- | -- | -- | .11 | -- | -- | -- | .31 | .06 | .05 | .07 | .29 | .12 | .03 | .10 |
| **Temporal, superior** | .15 | .04 | .00 | .10 | .19 | .05 | .00 | .11 | .53 | .34 | .09 | .56 | .57 | .36 | .11 | .56 |
| **Temporal, middle** | .17 | .04 | .01 | .04 | .19 | .04 | .01 | .05 | .51 | .40 | .19 | .49 | .54 | .40 | .19 | .48 |
| **Temporal, inferior** | .17 | .02 | .02 | .02 | .17 | .02 | .01 | .03 | .48 | .28 | .18 | .35 | .48 | .27 | .16 | .32 |
| **Temporal, transverse** | .02 | .00 | .01 | .00 | .03 | .00 | .01 | .00 | .47 | .17 | .06 | .27 | .47 | .20 | .17 | .29 |
| **Temporal, bank sup temp sulc** | .09 | .02 | .01 | .03 | .13 | .02 | .01 | .03 | .47 | .19 | .08 | .32 | .49 | .28 | .13 | .36 |
| **Temporal, fusiform** | .17 | .03 | .01 | .05 | .16 | .03 | .01 | .06 | .48 | .27 | .19 | .30 | .48 | .23 | .14 | .31 |
| **Occipital, lateral** | .03 | .02 | .01 | .03 | .02 | .03 | .01 | .02 | .36 | .25 | .15 | .21 | .35 | .21 | .11 | .25 |
| **Occipital, pericalcarine** | .00 | .00 | .00 | .00 | .02 | .00 | .00 | .00 | .27 | .06 | .03 | .18 | .22 | .06 | .04 | .13 |
| **Occipital, lingual** | .00 | .01 | .01 | .01 | .00 | .01 | .01 | .00 | .27 | .18 | .09 | .30 | .26 | .19 | .09 | .30 |
| **Occipital, cuneus** | .00 | .01 | .01 | .00 | .00 | .00 | .01 | .00 | .27 | .12 | .08 | .18 | .27 | .12 | .08 | .19 |
