# Supplementary material for: Individual variations in ‘brain age’ relate to early-life factors more than to longitudinal brain change
Source: eLife. 2021 Nov 10;10:e69995. doi: 10.7554/eLife.69995 (PMC8580481; doi:10.7554/eLife.69995)
Supplement: Supplementary file 2. — List of subcortical features included in the brain age model and age variance explained in the UK Biobank and the Lifebrain training datasets. Vol = volume; Int = intensity; hemi = hemisphere. [file elife-69995-supp2.docx]

|  | **Vol** | **Int** | **Vol** | **Int** | **Vol** | **Int** | **Vol** | **Int** | **Vol** | **Int** | **Vol** | **Int** |
| --- | --- | --- | --- | --- | --- | --- | --- | --- | --- | --- | --- | --- |
|  | UK Biobank | | | | | | Lifebrain | | | | | |
|  | Left hemi | | Right hemi | | Bilateral | | Left hemi | | Right hemi | | Bilateral | |
| **Ventricular ROIs** | | | | | | | | | | | | |
| **3rd Ventricle** | -- | -- | -- | -- | .19 | .22 | -- | -- | -- | -- | .55 | .45 |
| **4th Ventricle** | -- | -- | -- | -- | .02 | .02 | -- | -- | -- | -- | .02 | .01 |
| **5th Ventricle** | -- | -- | -- | -- | .00 | .00 | -- | -- | -- | -- | .05 | .03 |
| **Inf lat vent** | .18 | .12 | .15 | .06 | -- | -- | .41 | .11 | .39 | .02 | -- | -- |
| **Lat vent** | .16 | .11 | .16 | .11 | -- | -- | .43 | .11 | .45 | .12 | -- | -- |
| **CSF total** | -- | -- | -- | -- | .06 | .12 | -- | -- | -- | -- | .21 | .23 |
| **Volumetric ROIs** | | | | | | | | | | | | |
| **Accumbens** | .18 | .11 | .12 | .15 | -- | -- | .45 | .31 | .41 | .38 | -- | -- |
| **Amygdala** | .11 | .01 | .06 | .05 | -- | -- | .33 | .21 | .25 | .31 | -- | -- |
| **Brainstem** | -- | -- | -- | -- | .00 | .13 | -- | -- | -- | -- | .07 | .06 |
| **Caudate** | .00 | .01 | .00 | .01 | -- | -- | .18 | .22 | .16 | .15 | -- | -- |
| **Cerebellum** | .01 | .01 | .01 | .01 | -- | -- | .35 | .02 | .32 | .01 | -- | -- |
| **Hippocampus** | .11 | .02 | .11 | .02 | -- | -- | .37 | .24 | .39 | .25 | -- | -- |
| **Pallidum** | .01 | .04 | .01 | .03 | -- | -- | .09 | .10 | .04 | .12 | -- | -- |
| **Putamen** | .04 | .01 | .04 | .00 | -- | -- | .35 | .13 | .37 | .12 | -- | -- |
| **Thalamus** | .10 | .15 | .07 | .16 | -- | -- | .45 | .04 | .43 | .04 | -- | -- |
| **White matter** | | | | | | | | | | | | |
| **CC anterior** | -- | -- | -- | -- | .02 | .06 | -- | -- | -- | -- | .10 | .10 |
| **CC central** | -- | -- | -- | -- | .08 | .05 | -- | -- | -- | -- | .28 | .04 |
| **CC mid anterior** | -- | -- | -- | -- | .10 | .04 | -- | -- | -- | -- | .22 | .06 |
| **CC mid posterior** | -- | -- | -- | -- | .05 | .13 | -- | -- | -- | -- | .24 | .12 |
| **CC posterior** | -- | -- | -- | -- | .00 | .07 | -- | -- | -- | -- | .03 | .08 |
| **Cerebellum WM** | .07 | .00 | .06 | .00 | -- | -- | .08 | .02 | .10 | .02 | -- | -- |
| **Cerebral WM** | .03 | -- | .03 | -- | -- | -- | .15 | -- | .13 | -- | .14 | -- |
| **WM hypointensities** | -- | -- | -- | -- | .14 | .05 | -- | -- | -- | -- | .31 | .15 |
| **Non WM hypointensities** | -- | -- | -- | -- | .00 | .00 | -- | -- | -- | -- | .21 | .00 |
| **Global features** | | | | | | | | | | | | |
| **Brain Seg** | -- | -- | -- | -- | .02 | -- | -- | -- | -- | -- | .23 | -- |
| **Brain Seg not vent** | -- | -- | -- | -- | .05 | -- | -- | -- | -- | -- | .32 | -- |
| **Brain Seg not vent surf** | -- | -- | -- | -- | .05 | -- | -- | -- | -- | -- | .32 | -- |
| **Cortex** | .05 | -- | .05 | -- | -- | -- | .45 | -- | .44 | -- | .45 | -- |
| **eICV** | -- | -- | -- | -- | .00 | -- | -- | -- | -- | -- | .00 | -- |
| **Subcort gray** | -- | -- | -- | -- | .06 | -- | -- | -- | -- | -- | .44 | -- |
| **Supratentorial** | -- | -- | -- | -- | .02 | -- | -- | -- | -- | -- | .21 | -- |
| **Supratentorial not vent** | -- | -- | -- | -- | .05 | -- | -- | -- | -- | -- | .30 | -- |
| **Total gray** | -- | -- | -- | -- | .05 | -- | -- | -- | -- | -- | .47 | -- |
| **Other** | | | | | | | | | | | | |
| **Choroid plexus** | .18 | .17 | .18 | .18 | -- | -- | .35 | .33 | .34 | .32 | -- | -- |
| **Optic chiasm** | -- | -- | -- | -- | .05 | .00 | -- | -- | -- | -- | .09 | .01 |
| **Ventral DC** | .05 | .06 | .06 | .05 | -- | -- | .19 | .11 | .25 | .09 | -- | -- |
| **Ventricle choroid** | -- | -- | -- | -- | .18 | -- | -- | -- | -- | -- | -- | -- |
| **Vessel** | .00 | .00 | .01 | .00 | -- | -- | .02 | .02 | .03 | .01 | -- | -- |
