# Supplementary material for: Individual variations in ‘brain age’ relate to early-life factors more than to longitudinal brain change
Source: eLife. 2021 Nov 10;10:e69995. doi: 10.7554/eLife.69995 (PMC8580481; doi:10.7554/eLife.69995)
Supplement: Supplementary file 3. — Main sample descriptives for the training and test datasets. Obs = mean number of observations per participant (SD). Follow-up = mean time (years) between the first and the last MRI observation (SD). For the test datasets, age and age range refer to age at baseline. *AIBL does not belong to the Lifebrain consortium but was included to enrich the replication sample. [file elife-69995-supp3.docx]

|  | **Training dataset** | | | | **Test dataset** | | | | |
| --- | --- | --- | --- | --- | --- | --- | --- | --- | --- |
| **Cohort** | N | Age | Age Range | Sex (f:m) | N (Obs) | Age | Age Range | Sex (f:m) | Follow-up |
| **UK Biobank** | 38,682 | 64.4 (7.6) | 44.8 - 82.6 | 20,470:18,212 | 1,372 (2) | 63.4 (7.2) | 47.2 - 80.6 | 685:687 | 2.3 (.1) |
| **Lifebrain Total** | 1792 | 50.3 (21.8) | 18.0 - 94.4 | 1075:717 | 1,500 (2.8 [1.2]) | 56.9 (18.9) | 18.1 - 89.0 | 769:731 | 3.4 (2.2) |
| LCBC | 838 | 35.5 (16.5) | 18.0 - 93.4 | 563:275 | 556 (3.7 [1.5]) | 48.9 (20.7) | 18.1 - 85.4 | 1,229:848 | 4.8 (2.7) |
| Cam-CAN | 386 | 54.7 (18.7) | 18.6 - 87.4 | 196:190 | 255 (2) | 55.0 (18.1) | 19.3 - 89.0 | 262:248 | 1.4 (0.7) |
| Base-II | 126 | 59.8 (18.1) | 25.0 - 82.0 | 58:68 | 319 (2) | 62.0 (16.6) | 24.1 - 81.3 | 224:414 | 1.9 (0.7) |
| Betula | 139 | 64.8 (13.0) | 25.9 - 84.6 | 75:64 | 170 (2) | 59.8 (13.8) | 25.5 - 80.8 | 166:174 | 4.0 (0.2) |
| UB | 6 | 64.3 (11.8) | 43.5 - 77.7 | 2:4 | 80 (2.7 [.4]) | 67.3 (6.9) | 36.8 - 78.1 | 135:84 | 3.7 (0.9) |
| AIBL* | 297 | 75.1 (5.7) | 61.8 - 94.4 | 181:116 | 120 (3.4 [.8]) | 73.0 (7.0) | 62.6 - 88.4 | 214:195 | 4.0 (1.4) |
