# Supplementary material for: Individual variations in ‘brain age’ relate to early-life factors more than to longitudinal brain change
Source: eLife. 2021 Nov 10;10:e69995. doi: 10.7554/eLife.69995 (PMC8580481; doi:10.7554/eLife.69995)
Supplement: Supplementary file 4. — Long. change = longitudinal change in the raw neuroimaging features (mean change [log10(p)]). PC1 load = feature loadings on the first component of longitudinal change. Deltacross = relationship between cross-sectional brain age delta and feature change (r2 [log10(p)]). Deltalong = relationship between longitudinal brain age delta and feature change (r2 [log10(p)]). GWC = gray-white matter contrast. Cth = cortical thickness. Bil = bilateral. Subc = subcortical. n = 1372 and 1500 for the UK Biobank and the Lifebrain datasets. |N| = 365 and 372 features in the UK Biobank and the Lifebrain datasets. XGB = boosting gradient as implemented in XGBoost. [file elife-69995-supp4.docx]

|  | | | **UK Biobank** | | | | | | **Lifebrain** | | | |
| --- | --- | --- | --- | --- | --- | --- | --- | --- | --- | --- | --- | --- |
| **Modality** | **Region** | **Hemi** | **Long. change** | **PC_1_ load** | ***delta*_Cross_ - XGB** | ***delta*_cross_ - LASSO** | **delta_long_ - XGB** | **delta_long_ - LASSO** | **Long. change** | **PC_1_ load** | ***delta*_cross_** | ***delta*_long_** |
| **Area** | **Total surface** | **Left** | **-.03 (45.5)** | **0.012** | **.001 (0.8)** | **.000 (0.1)** | **.001 (-0.5)** | **.002 (-1.1)** | **-.03 (68.3)** | **0.005** | **.000 (0.4)** | **.007 (3.0)** |
|  |  | **Right** | **-.03 (52.9)** | **0.004** | **.002 (0.8)** | **.000 (0.2)** | **.000 (-0.1)** | **.006 (-2.5)** | **-.03 (57.0)** | **0.006** | **.000 (-0.2)** | **.007 (-2.9)** |
|  | **Cingulate, caudal ant** | **Left** | **-.01 (1.5)** | **--** | **.003 (1.2)** | **.000 (0.2)** | **.000 (0.3)** | **.000 (0.0)** | **-.01 (14.6)** | **0.009** | **.000 (-0.1)** | **.025 (-9.1)** |
|  |  | **Right** | **-.01 (1.0)** | **--** | **.000 (0.0)** | **.001 (0.8)** | **.008 (-3.2)** | **.001 (-0.7)** | **-.02 (31.9)** | **0.008** | **.002 (-1.1)** | **.010 (-3.9)** |
|  | **Cingulate, rostral ant** | **Left** | **-.01 (1.2)** | **--** | **.000 (-0.3)** | **.000 (-0.2)** | **.000 (0.0)** | **.000 (0.3)** | **-.02 (10.3)** | **0.008** | **.001 (0.9)** | **.009 (3.7)** |
|  |  | **Right** | **-.01 (3.0)** | **--** | **.001 (0.7)** | **.002 (0.9)** | **.010 (-3.7)** | **.001 (-0.6)** | **-.01 (5.9)** | **0.011** | **.002 (-0.9)** | **.011 (-4.3)** |
|  | **Cingulate, posterior** | **Left** | **-.02 (4.8)** | **0.016** | **.001 (0.7)** | **.000 (0.0)** | **.003 (1.4)** | **.001 (0.5)** | **-.03 (49.8)** | **0.011** | **.000 (0.0)** | **.011 (4.2)** |
|  |  | **Right** | **-.01 (3.4)** | **--** | **.000 (0.1)** | **.001 (0.5)** | **.007 (-2.9)** | **.001 (-0.6)** | **-.03 (76.5)** | **0.011** | **.002 (-0.9)** | **.010 (-4.1)** |
|  | **Cingulate, isthmus** | **Left** | **-.01 (2.0)** | **--** | **.000 (0.0)** | **.000 (0.2)** | **.000 (0.2)** | **.005 (1.9)** | **-.02 (10.9)** | **0.003** | **.001 (-0.6)** | **.001 (-0.7)** |
|  |  | **Right** | **-.01 (2.5)** | **--** | **.000 (0.4)** | **.000 (0.3)** | **.001 (0.8)** | **.001 (0.5)** | **-.02 (10.3)** | **0.011** | **.000 (0.2)** | **.005 (2.3)** |
|  | **Insula** | **Left** | **-.00 (0.0)** | **--** | **.000 (0.3)** | **.000 (0.0)** | **.002 (-0.9)** | **.001 (-0.8)** | **-.01 (2.9)** | **--** | **.000 (-0.2)** | **.004 (-1.7)** |
|  |  | **Right** | **-.02 (1.8)** | **--** | **.001 (-0.5)** | **.000 (-0.2)** | **.000 (-0.1)** | **.000 (-0.1)** | **-.00 (0.6)** | **--** | **.000 (-0.3)** | **.005 (-2.1)** |
|  | **Frontal, superior** | **Left** | **-.01 (7.9)** | **0.006** | **.005 (1.9)** | **.001 (0.5)** | **.000 (-0.2)** | **.000 (-0.3)** | **-.02 (15.3)** | **-0.001** | **.000 (0.2)** | **.008 (3.2)** |
|  |  | **Right** | **-.01 (3.8)** | **--** | **.006 (2.3)** | **.001 (0.5)** | **.002 (-0.9)** | **.001 (-0.4)** | **-.01 (14.7)** | **-0.001** | **.000 (0.0)** | **.011 (4.5)** |
|  | **Frontal, caudal middle** | **Left** | **-.01 (3.2)** | **--** | **.002 (0.9)** | **.001 (0.4)** | **.001 (0.6)** | **.000 (0.1)** | **-.01 (7.7)** | **-0.006** | **.000 (-0.1)** | **.001 (-0.9)** |
|  |  | **Right** | **-.01 (1.5)** | **--** | **.001 (0.6)** | **.000 (0.2)** | **.000 (-0.2)** | **.002 (-1.0)** | **-.01 (6.8)** | **-0.004** | **.000 (-0.4)** | **.001 (-0.6)** |
|  | **Frontal, rostral middle** | **Left** | **-.02 (11.8)** | **0.011** | **.001 (0.6)** | **.000 (0.3)** | **.000 (0.0)** | **.003 (1.4)** | **-.02 (22.1)** | **0.006** | **.000 (-0.1)** | **.009 (-3.6)** |
|  |  | **Right** | **-.01 (3.2)** | **--** | **.002 (1.1)** | **.000 (0.1)** | **.005 (-2.1)** | **.002 (-1.2)** | **-.01 (12.8)** | **0.009** | **.001 (-0.7)** | **.037 (-13.4)** |
|  | **Frontal, pars opercularis** | **Left** | **-.02 (8.3)** | **0.007** | **.001 (0.7)** | **.000 (0.3)** | **.000 (0.4)** | **.000 (0.4)** | **-.02 (26.5)** | **0.001** | **.000 (-0.3)** | **.001 (-0.6)** |
|  |  | **Right** | **-.02 (6.6)** | **0.002** | **.000 (0.1)** | **.001 (0.7)** | **.001 (0.5)** | **.002 (0.9)** | **-.01 (12.5)** | **0.002** | **.000 (0.0)** | **.006 (2.6)** |
|  | **Frontal, pars triangularis** | **Left** | **-.04 (16.4)** | **0.010** | **.000 (0.3)** | **.000 (0.0)** | **.000 (0.0)** | **.002 (1.0)** | **-.02 (14.7)** | **0.005** | **.000 (0.4)** | **.009 (3.6)** |
|  |  | **Right** | **-.02 (8.2)** | **0.003** | **.000 (0.3)** | **.001 (0.5)** | **.005 (-2.0)** | **.007 (-2.6)** | **-.02 (10.3)** | **0.011** | **.000 (-0.1)** | **.017 (-6.2)** |
|  | **Frontal, pars orbitalis** | **Left** | **-.03 (18.5)** | **0.021** | **.000 (0.1)** | **.000 (0.1)** | **.002 (1.0)** | **.002 (0.9)** | **-.02 (34.4)** | **0.001** | **.001 (0.4)** | **.002 (0.9)** |
|  |  | **Right** | **-.02 (6.6)** | **0.004** | **.001 (0.5)** | **.000 (0.1)** | **.000 (0.1)** | **.000 (0.2)** | **-.02 (23.7)** | **0.004** | **.000 (-0.2)** | **.005 (-2.3)** |
|  | **Frontal, lateral orbital** | **Left** | **-.03 (10.2)** | **0.016** | **.000 (0.0)** | **.000 (0.0)** | **.013 (-4.7)** | **.026 (-9.0)** | **-.02 (15.4)** | **0.019** | **.001 (-0.7)** | **.024 (-8.7)** |
|  |  | **Right** | **-.02 (1.5)** | **--** | **.001 (-0.4)** | **.000 (-0.3)** | **.009 (-3.5)** | **.009 (-3.5)** | **-.01 (2.0)** | **--** | **.000 (-0.1)** | **.003 (-1.3)** |
|  | **Frontal, medial orbital** | **Left** | **-.02 (2.9)** | **--** | **.000 (0.0)** | **.001 (0.4)** | **.000 (0.1)** | **.001 (0.4)** | **-.02 (2.7)** | **--** | **.000 (0.1)** | **.006 (2.6)** |
|  |  | **Right** | **-.01 (1.1)** | **--** | **.000 (0.0)** | **.000 (0.0)** | **.001 (-0.7)** | **.003 (-1.3)** | **-.01 (1.5)** | **--** | **.000 (-0.2)** | **.014 (-5.4)** |
|  | **Frontal, pole** | **Left** | **-.04 (7.7)** | **0.019** | **.000 (0.1)** | **.001 (0.6)** | **.000 (0.0)** | **.002 (0.9)** | **-.06 (15.7)** | **0.027** | **.000 (-0.2)** | **.018 (-6.9)** |
|  |  | **Right** | **-.01 (0.7)** | **--** | **.000 (-0.2)** | **.001 (-0.7)** | **.001 (0.7)** | **.001 (0.5)** | **-.04 (7.9)** | **0.021** | **.000 (0.0)** | **.007 (3.1)** |
|  | **Frontal, precentral gyrus** | **Left** | **-.01 (3.2)** | **--** | **.001 (0.5)** | **.000 (0.2)** | **.000 (0.0)** | **.002 (0.9)** | **-.01 (1.5)** | **--** | **.001 (0.4)** | **.005 (2.2)** |
|  |  | **Right** | **-.00 (0.7)** | **--** | **.001 (0.5)** | **.000 (0.3)** | **.000 (0.1)** | **.007 (2.7)** | **-.01 (2.0)** | **--** | **.000 (0.1)** | **.015 (5.7)** |
|  | **Parietal, postcentral gyrus** | **Left** | **-.01 (8.4)** | **-0.004** | **.000 (0.2)** | **.000 (0.1)** | **.000 (0.1)** | **.001 (0.6)** | **-.01 (6.7)** | **-0.014** | **.001 (0.5)** | **.005 (2.1)** |
|  |  | **Right** | **-.01 (5.0)** | **-0.007** | **.001 (-0.7)** | **.000 (-0.2)** | **.005 (2.1)** | **.001 (0.7)** | **-.01 (3.4)** | **--** | **.001 (0.5)** | **.014 (5.3)** |
|  | **Parietal, paracentral gyrus** | **Left** | **-.00 (0.3)** | **--** | **.002 (0.9)** | **.002 (0.9)** | **.001 (0.6)** | **.001 (0.8)** | **-.01 (2.8)** | **--** | **.001 (0.6)** | **.001 (0.8)** |
|  |  | **Right** | **-.01 (1.0)** | **--** | **.000 (-0.1)** | **.000 (-0.4)** | **.001 (-0.7)** | **.000 (-0.3)** | **-.01 (2.1)** | **--** | **.000 (0.0)** | **.007 (3.0)** |
|  | **Parietal, superior** | **Left** | **-.02 (20.4)** | **0.005** | **.000 (-0.2)** | **.002 (-1.1)** | **.000 (0.2)** | **.005 (1.9)** | **-.02 (21.8)** | **-0.002** | **.000 (-0.2)** | **.001 (-0.6)** |
|  |  | **Right** | **-.02 (8.0)** | **0.005** | **.001 (0.6)** | **.000 (0.1)** | **.007 (2.7)** | **.000 (0.2)** | **-.02 (20.4)** | **-0.004** | **.000 (-0.1)** | **.001 (-0.6)** |
|  | **Parietal, inferior** | **Left** | **-.03 (33.1)** | **0.017** | **.001 (0.5)** | **.002 (0.9)** | **.001 (0.7)** | **.004 (1.8)** | **-.02 (62.1)** | **0.005** | **.000 (-0.1)** | **.006 (-2.6)** |
|  |  | **Right** | **-.03 (29.0)** | **0.004** | **.000 (0.2)** | **.000 (0.3)** | **.000 (-0.2)** | **.000 (-0.2)** | **-.02 (53.3)** | **0.003** | **.000 (0.0)** | **.000 (0.1)** |
|  | **Parietal, supramarginal** | **Left** | **-.02 (13.9)** | **0.008** | **.000 (-0.1)** | **.001 (-0.8)** | **.001 (0.4)** | **.001 (0.6)** | **-.02 (40.8)** | **0.001** | **.000 (0.2)** | **.002 (1.2)** |
|  |  | **Right** | **-.01 (4.6)** | **-0.005** | **.001 (-0.6)** | **.000 (-0.1)** | **.000 (0.3)** | **.000 (0.2)** | **-.02 (33.1)** | **-0.002** | **.000 (0.0)** | **.001 (0.6)** |
|  | **Parietal, precuneus** | **Left** | **-.02 (17.1)** | **0.013** | **.001 (0.4)** | **.001 (0.6)** | **.006 (2.5)** | **.001 (0.6)** | **-.02 (55.8)** | **0.004** | **.000 (0.4)** | **.004 (1.7)** |
|  |  | **Right** | **-.02 (12.9)** | **0.013** | **.002 (1.2)** | **.000 (0.2)** | **.009 (3.4)** | **.001 (0.6)** | **-.02 (49.4)** | **0.001** | **.000 (-0.2)** | **.000 (-0.3)** |
|  | **Temporal, parahippocampal** | **Left** | **-.03 (6.9)** | **-0.009** | **.001 (-0.5)** | **.001 (-0.4)** | **.004 (-1.6)** | **.004 (-1.8)** | **-.02 (14.1)** | **0.004** | **.005 (2.1)** | **.002 (0.9)** |
|  |  | **Right** | **-.03 (7.8)** | **0.001** | **.000 (0.0)** | **.000 (0.2)** | **.000 (0.0)** | **.000 (0.4)** | **-.02 (15.4)** | **0.013** | **.003 (-1.3)** | **.012 (-4.7)** |
|  | **Temporal, entorhinal** | **Left** | **-.00 (0.2)** | **--** | **.000 (0.2)** | **.001 (0.5)** | **.001 (0.5)** | **.000 (0.2)** | **-.02 (5.2)** | **0.003** | **.000 (0.3)** | **.001 (0.4)** |
|  |  | **Right** | **-.01 (0.4)** | **--** | **.002 (1.1)** | **.002 (0.9)** | **.001 (0.4)** | **.000 (0.0)** | **-.01 (2.5)** | **--** | **.001 (-0.5)** | **.008 (-3.5)** |
|  | **Temporal, pole** | **Left** | **--** | **--** | **--** | **--** | **--** | **--** | **-.03 (6.0)** | **0.021** | **.001 (0.5)** | **.001 (0.6)** |
|  |  | **Right** | **--** | **--** | **--** | **--** | **--** | **--** | **-.03 (7.3)** | **0.035** | **.000 (-0.1)** | **.007 (-2.9)** |
|  | **Temporal, superior** | **Left** | **-.02 (16.2)** | **0.009** | **.000 (0.3)** | **.000 (0.2)** | **.000 (-0.1)** | **.003 (-1.2)** | **-.02 (56.6)** | **0.006** | **.000 (0.2)** | **.000 (0.2)** |
|  |  | **Right** | **-.02 (20.0)** | **-0.005** | **.000 (-0.1)** | **.000 (-0.2)** | **.000 (-0.1)** | **.002 (-0.9)** | **-.02 (33.4)** | **0.004** | **.001 (-0.8)** | **.000 (-0.1)** |
|  | **Temporal, middle** | **Left** | **-.04 (45.0)** | **0.012** | **.000 (0.1)** | **.000 (0.1)** | **.002 (-1.0)** | **.001 (-0.5)** | **-.03 (71.2)** | **0.012** | **.001 (0.5)** | **.011 (4.3)** |
|  |  | **Right** | **-.03 (38.0)** | **0.007** | **.000 (-0.3)** | **.000 (-0.3)** | **.006 (-2.6)** | **.003 (-1.3)** | **-.03 (60.7)** | **0.011** | **.000 (-0.1)** | **.006 (-2.8)** |
|  | **Temporal, inferior** | **Left** | **-.04 (42.0)** | **0.015** | **.000 (-0.1)** | **.000 (-0.4)** | **.012 (-4.3)** | **.009 (-3.5)** | **-.02 (46.5)** | **0.017** | **.000 (-0.3)** | **.052 (-18.7)** |
|  |  | **Right** | **-.03 (31.2)** | **0.007** | **.000 (0.1)** | **.001 (0.6)** | **.003 (-1.3)** | **.007 (-2.7)** | **-.03 (54.4)** | **0.015** | **.000 (-0.2)** | **.038 (-13.7)** |
|  | **Temporal, transverse** | **Left** | **-.02 (4.8)** | **0.005** | **.001 (0.4)** | **.001 (0.4)** | **.004 (1.6)** | **.000 (0.0)** | **-.03 (18.4)** | **0.007** | **.000 (0.3)** | **.001 (0.6)** |
|  |  | **Right** | **-.01 (0.8)** | **--** | **.000 (-0.4)** | **.001 (-0.5)** | **.000 (0.1)** | **.000 (0.3)** | **-.05 (25.5)** | **0.001** | **.000 (-0.1)** | **.001 (-0.5)** |
|  | **Temporal, bank sup temp sulc** | **Left** | **-.03 (19.4)** | **0.007** | **.000**  **(0.0)** | **.000 (0.0)** | **.000 (-0.2)** | **.001 (-0.7)** | **-.01 (21.5)** | **0.001** | **.002 (-1.3)** | **.005 (-2.3)** |
|  |  | **Right** | **-.03 (16.4)** | **-0.003** | **.000**  **(0.0)** | **.000 (0.2)** | **.001 (-0.6)** | **.000 (-0.3)** | **-.02 (37.9)** | **0.002** | **.006 (-2.9)** | **.001 (-0.8)** |
|  | **Temporal, fusiform** | **Left** | **-.04 (31.8)** | **0.013** | **.001**  **(0.7)** | **.001 (0.7)** | **.003 (-1.5)** | **.006 (-2.4)** | **-.03 (55.2)** | **0.014** | **.000 (0.0)** | **.005 (2.4)** |
|  |  | **Right** | **-.04 (28.8)** | **0.017** | **.003**  **(1.5)** | **.001 (0.5)** | **.000 (-0.3)** | **.006 (-2.4)** | **-.02 (38.6)** | **0.014** | **.000 (-0.1)** | **.011 (-4.4)** |
|  | **Occipital, lateral** | **Left** | **-.03 (29.5)** | **0.021** | **.000**  **(0.3)** | **.000 (0.2)** | **.003 (1.3)** | **.007 (2.7)** | **-.03 (57.4)** | **0.009** | **.000 (0.4)** | **.000 (0.3)** |
|  |  | **Right** | **-.03 (35.7)** | **0.015** | **.000**  **(0.3)** | **.001 (0.6)** | **.002 (1.1)** | **.003 (1.5)** | **-.03 (41.8)** | **0.009** | **.000 (0.3)** | **.002 (0.9)** |
|  | **Occipital, pericalcarine** | **Left** | **-.01 (6.4)** | **0.017** | **.006**  **(2.3)** | **.001 (0.4)** | **.000 (0.3)** | **.003 (1.4)** | **-.02 (20.1)** | **0.008** | **.002 (1.0)** | **.001 (0.7)** |
|  |  | **Right** | **-.02 (11.9)** | **0.013** | **.002**  **(0.8)** | **.000 (0.2)** | **.001 (0.5)** | **.000 (0.2)** | **-.01 (6.7)** | **0.008** | **.003 (1.5)** | **.003 (1.4)** |
|  | **Occipital, lingual** | **Left** | **-.03 (29.3)** | **0.016** | **.000**  **(0.3)** | **.000 (0.2)** | **.010 (-3.6)** | **.006 (-2.4)** | **-.03 (38.9)** | **0.012** | **.002 (1.1)** | **.017 (6.4)** |
|  |  | **Right** | **-.03 (27.0)** | **0.018** | **.000**  **(-0.2)** | **.002 (-1.0)** | **.001 (0.5)** | **.001 (0.7)** | **-.03 (28.6)** | **0.014** | **.000 (0.0)** | **.025 (9.2)** |
|  | **Occipital, cuneus** | **Left** | **-.02 (14.9)** | **0.016** | **.000**  **(0.1)** | **.001 (0.6)** | **.003 (1.2)** | **.002 (0.8)** | **-.02 (20.7)** | **0.009** | **.001 (0.8)** | **.013 (5.0)** |
|  |  | **Right** | **-.02 (8.8)** | **0.017** | **.000**  **(-0.2)** | **.002 (-1.0)** | **.002 (1.0)** | **.003 (1.4)** | **-.02 (12.5)** | **0.010** | **.000 (-0.3)** | **.003 (-1.3)** |
| **Cth** | **Total surface** | **Left** | **-.05 (8.2)** | **0.149** | **.000 (-0.1)** | **.001 (-0.8)** | **.004 (-1.8)** | **.005 (-2.0)** | **-.06 (40.7)** | **-0.001** | **.002 (-1.0)** | **.033 (-12.2)** |
|  |  | **Right** | **-.04 (5.3)** | **0.126** | **.000 (0.0)** | **.004 (1.9)** | **.000 (-0.2)** | **.000 (-0.3)** | **-.07 (38.0)** | **0.000** | **.002 (-1.0)** | **.031 (-11.4)** |
|  | **Cingulate, caudal ant** | **Left** | **-.01 (0.6)** | **--** | **.001 (0.7)** | **.001 (0.5)** | **.000 (-0.3)** | **.002 (-1.0)** | **-.03 (9.4)** | **-0.026** | **.000 (-0.4)** | **.000 (-0.0)** |
|  |  | **Right** | **-.02 (1.0)** | **--** | **.000 (-0.2)** | **.002 (-1.2)** | **.000 (0.3)** | **.000 (0.3)** | **-.01 (1.1)** | **--** | **.000 (-0.2)** | **.001 (-0.9)** |
|  | **Cingulate, rostral ant** | **Left** | **-.04 (5.1)** | **0.086** | **.000 (0.0)** | **.000 (0.2)** | **.001 (-0.5)** | **.003 (-1.3)** | **-.03 (6.8)** | **-0.003** | **.002 (-1.0)** | **.004 (-1.8)** |
|  |  | **Right** | **-.02 (1.0)** | **--** | **.004 (-1.7)** | **.006 (-2.3)** | **.000 (0.2)** | **.000 (0.3)** | **-.01 (1.5)** | **--** | **.000 (-0.2)** | **.004 (-1.8)** |
|  | **Cingulate, posterior** | **Left** | **-.04 (4.1)** | **0.088** | **.001 (-0.7)** | **.002 (-1.0)** | **.000 (0.0)** | **.001 (0.6)** | **-.05 (27.2)** | **-0.015** | **.002 (-1.0)** | **.001 (-0.5)** |
|  |  | **Right** | **-.02 (1.1)** | **--** | **.000 (-0.2)** | **.002 (-0.9)** | **.000 (-0.1)** | **.000 (-0.4)** | **-.03 (11.6)** | **-0.019** | **.000 (0.2)** | **.000 (0.3)** |
|  | **Cingulate, isthmus** | **Left** | **-.02 (2.0)** | **--** | **.000 (0.1)** | **.000 (0.1)** | **.002 (-1.0)** | **.002 (-1.1)** | **-.04 (26.4)** | **0.007** | **.000 (-0.1)** | **.022 (-8.2)** |
|  |  | **Right** | **-.01 (1.3)** | **--** | **.001 (-0.4)** | **.005 (-2.0)** | **.000 (0.0)** | **.006 (2.3)** | **-.03 (13.7)** | **0.005** | **.000 (-0.1)** | **.024 (-8.6)** |
|  | **Insula** | **Left** | **-.04 (3.7)** | **--** | **.001 (-0.7)** | **.001 (-0.6)** | **.000 (-0.3)** | **.000 (-0.1)** | **-.05 (23.7)** | **0.004** | **.001 (-0.4)** | **.018 (-6.8)** |
|  |  | **Right** | **-.01 (0.5)** | **--** | **.000 (0.0)** | **.000 (0.4)** | **.000 (-0.1)** | **.000 (-0.0)** | **-.05 (23.6)** | **-0.007** | **.001 (-0.7)** | **.000 (-0.1)** |
|  | **Frontal, superior** | **Left** | **-.05 (9.0)** | **0.109** | **.001 (-0.8)** | **.003 (-1.5)** | **.002 (-1.0)** | **.010 (-3.8)** | **-.05 (19.5)** | **-0.037** | **.001 (-0.6)** | **.005 (-2.1)** |
|  |  | **Right** | **-.04 (5.6)** | **0.096** | **.001 (-0.6)** | **.009 (-3.3)** | **.001 (0.5)** | **.003 (1.4)** | **-.04 (13.8)** | **-0.040** | **.002 (-1.3)** | **.004 (-1.7)** |
|  | **Frontal, caudal middle** | **Left** | **-.02 (1.8)** | **--** | **.000 (0.0)** | **.001 (0.7)** | **.002 (-0.9)** | **.004 (-1.8)** | **-.06 (18.0)** | **-0.014** | **.002 (-1.2)** | **.017 (-6.3)** |
|  |  | **Right** | **-.03 (3.0)** | **--** | **.000 (-0.1)** | **.003 (-1.5)** | **.000 (0.1)** | **.000 (0.3)** | **-.05 (11.4)** | **-0.022** | **.001 (-0.7)** | **.007 (-3.0)** |
|  | **Frontal, rostral middle** | **Left** | **-.05 (10.7)** | **0.109** | **.001 (-0.5)** | **.003 (-1.4)** | **.003 (-1.2)** | **.011 (-4.2)** | **-.05 (12.7)** | **-0.023** | **.002 (-1.0)** | **.015 (-5.8)** |
|  |  | **Right** | **-.04 (7.3)** | **0.102** | **.000 (-0.1)** | **.006 (-2.4)** | **.000 (-0.1)** | **.003 (-1.2)** | **-.05 (11.8)** | **-0.033** | **.000 (-0.2)** | **.010 (-4.1)** |
|  | **Frontal, pars opercularis** | **Left** | **-.04 (5.0)** | **0.107** | **.000 (0.0)** | **.002 (0.9)** | **.005 (-2.1)** | **.011 (-4.1)** | **-.04 (14.4)** | **-0.010** | **.001 (-0.6)** | **.010 (-4.2)** |
|  |  | **Right** | **-.02 (3.0)** | **--** | **.000 (0.2)** | **.001 (0.7)** | **.000 (-0.3)** | **.000 (-0.4)** | **-.05 (18.3)** | **-0.012** | **.000 (-0.3)** | **.011 (-4.3)** |
|  | **Frontal, pars triangularis** | **Left** | **-.02 (2.0)** | **--** | **.000 (-0.2)** | **.001 (-0.6)** | **.002 (-0.9)** | **.005 (-2.2)** | **-.04 (9.6)** | **-0.010** | **.000 (-0.1)** | **.013 (-5.0)** |
|  |  | **Right** | **-.00 (0.1)** | **--** | **.001 (0.6)** | **.002 (0.9)** | **.000 (0.2)** | **.000 (0.4)** | **-.04 (11.9)** | **-0.011** | **.000 (0.1)** | **.002 (1.3)** |
|  | **Frontal, pars orbitalis** | **Left** | **-.03 (3.7)** | **--** | **.000 (0.2)** | **.000 (0.3)** | **.003 (-1.5)** | **.004 (-1.8)** | **-.04 (13.7)** | **-0.020** | **.001 (-0.8)** | **.011 (-4.3)** |
|  |  | **Right** | **-.02 (2.6)** | **--** | **.000 (0.3)** | **.001 (0.6)** | **.002 (-0.9)** | **.006 (-2.4)** | **-.05 (18.6)** | **-0.008** | **.001 (-0.5)** | **.017 (-6.5)** |
|  | **Frontal, lateral orbital** | **Left** | **-.03 (2.0)** | **--** | **.000 (-0.2)** | **.002 (-1.0)** | **.000 (0.2)** | **.003 (1.4)** | **-.05 (17.5)** | **0.006** | **.000 (0.3)** | **.034 (12.6)** |
|  |  | **Right** | **-.00 (0.1)** | **--** | **.001 (0.5)** | **.001 (0.5)** | **.006 (2.3)** | **.008 (3.2)** | **-.04 (6.6)** | **-0.017** | **.002 (-0.9)** | **.022 (-8.1)** |
|  | **Frontal, medial orbital** | **Left** | **-.06 (7.4)** | **0.103** | **.000 (0.2)** | **.000 (0.1)** | **.000 (-0.4)** | **.000 (-0.0)** | **-.07 (17.4)** | **0.010** | **.000 (-0.2)** | **.003 (-1.5)** |
|  |  | **Right** | **-.06 (8.1)** | **0.091** | **.000 (-0.3)** | **.007 (-2.7)** | **.001 (0.7)** | **.001 (0.8)** | **-.04 (8.2)** | **-0.018** | **.000 (-0.3)** | **.020 (-7.4)** |
|  | **Frontal, pole** | **Left** | **-.03 (2.7)** | **--** | **.000 (0.0)** | **.000 (0.4)** | **.000 (-0.2)** | **.000 (-0.4)** | **-.03 (6.1)** | **-0.018** | **.000 (0.1)** | **.002 (1.1)** |
|  |  | **Right** | **-.03 (2.9)** | **--** | **.000 (-0.4)** | **.005 (-2.0)** | **.001 (0.7)** | **.000 (0.0)** | **-.03 (3.7)** | **--** | **.000 (0.2)** | **.003 (1.6)** |
|  | **Frontal, precentral gyrus** | **Left** | **-.03 (3.2)** | **--** | **.000 (-0.2)** | **.001 (-0.8)** | **.008 (-2.9)** | **.008 (-3.0)** | **-.06 (13.7)** | **0.006** | **.003 (-1.4)** | **.012 (-4.7)** |
|  |  | **Right** | **-.04 (4.4)** | **0.112** | **.000 (0.0)** | **.003 (1.2)** | **.001 (-0.7)** | **.001 (-0.8)** | **-.06 (12.2)** | **0.015** | **.002 (-1.0)** | **.019 (-7.1)** |
|  | **Parietal, postcentral gyrus** | **Left** | **-.03 (3.6)** | **--** | **.000 (0.1)** | **.000 (0.3)** | **.008 (-3.0)** | **.006 (-2.4)** | **-.05 (12.1)** | **0.005** | **.002 (-1.0)** | **.010 (-4.0)** |
|  |  | **Right** | **-.03 (2.6)** | **--** | **.002 (0.9)** | **.001 (0.6)** | **.002 (-1.0)** | **.000 (-0.3)** | **-.05 (18.3)** | **0.007** | **.001 (-0.8)** | **.024 (-8.9)** |
|  | **Parietal, paracentral gyrus** | **Left** | **-.05 (7.2)** | **0.122** | **.000 (0.0)** | **.001 (0.5)** | **.008 (-3.0)** | **.003 (-1.4)** | **-.05 (10.8)** | **-0.013** | **.001 (-0.7)** | **.012 (-4.6)** |
|  |  | **Right** | **-.05 (9.2)** | **0.109** | **.000 (0.0)** | **.002 (0.9)** | **.000 (-0.3)** | **.000 (-0.0)** | **-.05 (8.0)** | **-0.020** | **.001 (-0.8)** | **.021 (-7.8)** |
|  | **Parietal, superior** | **Left** | **-.03 (4.3)** | **0.121** | **.000 (-0.1)** | **.001 (-0.7)** | **.003 (-1.3)** | **.002 (-1.0)** | **-.05 (11.2)** | **-0.003** | **.000 (-0.3)** | **.019 (-7.1)** |
|  |  | **Right** | **-.04 (4.7)** | **0.101** | **.000 (-0.3)** | **.006 (-2.3)** | **.001 (-0.8)** | **.001 (-0.5)** | **-.05 (11.8)** | **0.002** | **.000 (-0.4)** | **.011 (-4.1)** |
|  | **Parietal, inferior** | **Left** | **-.04 (7.4)** | **0.119** | **.000 (0.0)** | **.001 (0.8)** | **.005 (-2.1)** | **.006 (-2.3)** | **-.05 (12.7)** | **-0.002** | **.000 (-0.2)** | **.013 (-5.0)** |
|  |  | **Right** | **-.04 (6.9)** | **0.097** | **.001 (-0.4)** | **.006 (-2.3)** | **.000 (-0.1)** | **.001 (-0.5)** | **-.06 (18.9)** | **0.007** | **.001 (-0.6)** | **.014 (-5.3)** |
|  | **Parietal, supramarginal** | **Left** | **-.04 (6.2)** | **0.103** | **.002 (-1.0)** | **.003 (-1.5)** | **.004 (-1.6)** | **.003 (-1.5)** | **-.05 (21.7)** | **0.000** | **.000 (-0.4)** | **.022 (-8.2)** |
|  |  | **Right** | **-.04 (5.5)** | **0.096** | **.000 (0.1)** | **.001 (0.7)** | **.000 (-0.1)** | **.000 (-0.3)** | **-.06 (24.8)** | **0.006** | **.000 (-0.3)** | **.020 (-7.6)** |
|  | **Parietal, precuneus** | **Left** | **-.03 (5.7)** | **0.102** | **.000 (-0.2)** | **.002 (-0.9)** | **.004 (-1.8)** | **.004 (-1.6)** | **-.07 (29.2)** | **0.003** | **.000 (-0.3)** | **.011 (-4.2)** |
|  |  | **Right** | **-.03 (4.7)** | **0.080** | **.000 (-0.1)** | **.004 (-1.8)** | **.004 (-1.8)** | **.000 (-0.0)** | **-.06 (23.4)** | **0.003** | **.000 (-0.4)** | **.016 (-5.9)** |
|  | **Temporal, parahippocampal** | **Left** | **-.03 (3.1)** | **--** | **.000 (-0.2)** | **.000 (-0.1)** | **.001 (-0.6)** | **.000 (-0.2)** | **-.03 (11.7)** | **0.015** | **.003 (-1.4)** | **.023 (-8.4)** |
|  |  | **Right** | **-.01 (1.0)** | **--** | **.000 (0.0)** | **.001 (0.4)** | **.000 (0.1)** | **.000 (0.3)** | **-.03 (9.4)** | **0.014** | **.000 (-0.1)** | **.029 (-10.5)** |
|  | **Temporal, entorhinal** | **Left** | **-.06 (6.7)** | **0.091** | **.002 (-0.8)** | **.001 (-0.5)** | **.000 (-0.2)** | **.000 (-0.0)** | **-.05 (14.0)** | **0.024** | **.000 (-0.1)** | **.008 (-3.3)** |
|  |  | **Right** | **-.04 (4.7)** | **0.067** | **.000 (-0.3)** | **.002 (-1.0)** | **.000 (0.0)** | **.001 (0.7)** | **-.03 (11.1)** | **0.025** | **.002 (-1.2)** | **.033 (-11.8)** |
|  | **Temporal, pole** | **Left** | **--** | **--** | **--** | **--** | **--** | **--** | **-.02 (3.7)** | **--** | **.000 (0.0)** | **.002 (1.2)** |
|  |  | **Right** | **--** | **--** | **--** | **--** | **--** | **--** | **-.04 (8.4)** | **-0.001** | **.000 (-0.4)** | **.009 (-3.8)** |
|  | **Temporal, superior** | **Left** | **-.05 (9.7)** | **0.125** | **.000 (0.0)** | **.000 (0.2)** | **.007 (-2.9)** | **.004 (-1.8)** | **-.05 (39.0)** | **0.004** | **.001 (-0.5)** | **.031 (-11.5)** |
|  |  | **Right** | **-.06 (14.7)** | **0.107** | **.000 (-0.1)** | **.003 (-1.2)** | **.002 (-1.1)** | **.006 (-2.4)** | **-.06 (48.2)** | **0.008** | **.000 (-0.4)** | **.032 (-12.3)** |
|  | **Temporal, middle** | **Left** | **-.06 (9.9)** | **0.139** | **.000 (0.1)** | **.000 (0.2)** | **.003 (-1.3)** | **.011 (-3.9)** | **-.05 (30.1)** | **0.001** | **.000 (-0.1)** | **.024 (-9.3)** |
|  |  | **Right** | **-.05 (5.7)** | **0.122** | **.000 (0.2)** | **.001 (0.8)** | **.000 (0.1)** | **.003 (1.4)** | **-.08 (46.9)** | **0.016** | **.001 (-0.5)** | **.032 (-12.3)** |
|  | **Temporal, inferior** | **Left** | **-.05 (6.8)** | **0.117** | **.000 (0.2)** | **.000 (0.1)** | **.002 (-0.9)** | **.006 (-2.3)** | **-.06 (26.3)** | **0.014** | **.000 (0.2)** | **.033 (12.6)** |
|  |  | **Right** | **-.04 (4.6)** | **0.099** | **.000 (0.0)** | **.003 (1.3)** | **.000 (0.2)** | **.001 (0.7)** | **-.07 (37.2)** | **0.019** | **.000 (-0.4)** | **.024 (-9.1)** |
|  | **Temporal, transverse** | **Left** | **-.01 (0.4)** | **--** | **.000 (0.0)** | **.001 (0.7)** | **.003 (-1.5)** | **.000 (-0.0)** | **-.04 (7.4)** | **-0.007** | **.000 (-0.4)** | **.000 (-0.1)** |
|  |  | **Right** | **-.02 (2.0)** | **0.081** | **.000 (-0.1)** | **.002 (-0.9)** | **.002 (-0.9)** | **.000 (-0.4)** | **-.02 (1.9)** | **--** | **.000 (0.2)** | **.006 (2.6)** |
|  | **Temporal, bank sup temp sulc** | **Left** | **-.03 (4.5)** | **0.063** | **.000 (-0.2)** | **.002 (-0.9)** | **.002 (-1.0)** | **.000 (-0.1)** | **-.05 (14.9)** | **0.012** | **.000 (0.0)** | **.008 (3.3)** |
|  |  | **Right** | **-.03 (5.1)** | **--** | **.000 (-0.2)** | **.003 (-1.3)** | **.001 (-0.4)** | **.000 (-0.0)** | **-.07 (26.7)** | **0.015** | **.000 (-0.1)** | **.007 (-3.0)** |
|  | **Temporal, fusiform** | **Left** | **-.07 (15.4)** | **0.121** | **.001 (-0.5)** | **.003 (-1.4)** | **.003 (-1.2)** | **.001 (-0.8)** | **-.07 (28.3)** | **0.038** | **.001 (-0.7)** | **.053 (-19.2)** |
|  |  | **Right** | **-.03 (2.5)** | **--** | **.000 (-0.2)** | **.003 (-1.3)** | **.000 (0.0)** | **.000 (0.1)** | **-.07 (30.6)** | **0.035** | **.001 (-0.8)** | **.026 (-9.7)** |
|  | **Occipital, lateral** | **Left** | **-.04 (5.6)** | **0.128** | **.000 (0.3)** | **.000 (0.3)** | **.001 (-0.6)** | **.001 (-0.8)** | **-.05 (13.9)** | **0.021** | **.001 (-0.8)** | **.014 (-5.4)** |
|  |  | **Right** | **-.01 (0.9)** | **--** | **.000 (0.3)** | **.002 (0.9)** | **.001 (-0.6)** | **.000 (-0.1)** | **-.06 (19.1)** | **0.025** | **.002 (-1.1)** | **.017 (-6.4)** |
|  | **Occipital, pericalcarine** | **Left** | **-.00 (0.0)** | **--** | **.001 (0.5)** | **.000 (0.1)** | **.001 (-0.4)** | **.002 (-0.8)** | **-.02 (2.3)** | **--** | **.001 (-0.5)** | **.000 (-0.3)** |
|  |  | **Right** | **-.01 (0.7)** | **--** | **.003 (1.3)** | **.001 (0.5)** | **.002 (1.0)** | **.004 (1.6)** | **-.04 (6.3)** | **0.009** | **.000 (-0.1)** | **.004 (-1.7)** |
|  | **Occipital, lingual** | **Left** | **-.00 (0.1)** | **--** | **.000 (0.2)** | **.000 (0.3)** | **.000 (-0.2)** | **.000 (-0.4)** | **-.05 (16.7)** | **0.022** | **.002 (-1.2)** | **.001 (-0.5)** |
|  |  | **Right** | **-.01 (0.4)** | **--** | **.002 (0.9)** | **.001 (0.4)** | **.000 (0.2)** | **.003 (1.2)** | **-.06 (16.3)** | **0.017** | **.000 (-0.2)** | **.000 (-0.4)** |
|  | **Occipital, cuneus** | **Left** | **-.01 (0.7)** | **--** | **.000 (0.3)** | **.000 (0.1)** | **.002 (-0.9)** | **.000 (-0.3)** | **-.03 (7.9)** | **0.007** | **.000 (-0.2)** | **.000 (-0.1)** |
|  |  | **Right** | **-.01 (0.8)** | **--** | **.001 (0.7)** | **.001 (0.4)** | **.001 (0.7)** | **.005 (2.1)** | **-.03 (5.0)** | **0.003** | **.000 (-0.1)** | **.001 (-0.4)** |
| **GWC** | **Cingulate, caudal ant** | **Left** | **-.06 (6.3)** | **0.137** | **.001 (0.7)** | **.001 (0.5)** | **.006 (-2.2)** | **.017 (-6.1)** | **-.04 (9.1)** | **0.157** | **.000 (-0.1)** | **.071 (-28.8)** |
|  |  | **Right** | **-.06 (7.8)** | **0.131** | **.000 (0.0)** | **.001 (0.5)** | **.003 (-1.5)** | **.010 (-3.6)** | **-.03 (5.3)** | **0.144** | **.001 (0.8)** | **.116 (46.4)** |
|  | **Cingulate, rostral ant** | **Left** | **-.08 (21.2)** | **0.080** | **.002 (0.9)** | **.001 (0.6)** | **.017 (-5.9)** | **.032 (-10.9)** | **-.05 (9.2)** | **0.135** | **.001 (-0.4)** | **.064 (-24.6)** |
|  |  | **Right** | **-.07 (16.0)** | **0.091** | **.000 (0.0)** | **.000 (0.0)** | **.010 (-3.9)** | **.010 (-3.8)** | **-.04 (7.1)** | **0.147** | **.000 (0.4)** | **.085 (33.3)** |
|  | **Cingulate, posterior** | **Left** | **-.10 (31.5)** | **0.118** | **.000 (-0.3)** | **.000 (-0.2)** | **.014 (-5.0)** | **.035 (-11.8)** | **-.04 (10.5)** | **0.143** | **.000 (0.4)** | **.079 (33.9)** |
|  |  | **Right** | **-.08 (22.5)** | **0.104** | **.000 (-0.1)** | **.000 (-0.4)** | **.013 (-4.6)** | **.035 (-12.0)** | **-.03 (5.0)** | **0.148** | **.000 (0.4)** | **.103 (42.5)** |
|  | **Cingulate, isthmus** | **Left** | **-.01 (0.7)** | **--** | **.001 (0.4)** | **.001 (0.5)** | **.003 (-1.3)** | **.005 (-2.2)** | **-.04 (8.5)** | **0.132** | **.000 (0.2)** | **.054 (23.1)** |
|  |  | **Right** | **-.02 (2.0)** | **--** | **.001 (-0.6)** | **.001 (-0.7)** | **.001 (-0.7)** | **.001 (-0.5)** | **-.03 (4.2)** | **0.134** | **.000 (0.0)** | **.058 (24.5)** |
|  | **Insula** | **Left** | **-.05 (10.2)** | **0.076** | **.000 (0.0)** | **.000 (0.1)** | **.000 (-0.2)** | **.002 (-0.9)** | **-.05 (16.0)** | **0.149** | **.001 (1.0)** | **.051 (22.2)** |
|  |  | **Right** | **-.02 (1.1)** | **--** | **.000 (-0.4)** | **.001 (-0.4)** | **.004 (-1.8)** | **.007 (-2.7)** | **-.01 (1.6)** | **--** | **.000 (0.1)** | **.042 (17.9)** |
|  | **Frontal, superior** | **Left** | **-.13 (70.6)** | **0.106** | **.000 (0.0)** | **.000 (0.1)** | **.046 (-15.9)** | **.053 (-18.1)** | **-.05 (21.5)** | **0.129** | **.001 (0.8)** | **.147 (60.1)** |
|  |  | **Right** | **-.12 (67.5)** | **0.104** | **.000 (0.1)** | **.000 (0.1)** | **.046 (-15.5)** | **.057 (-19.4)** | **-.03 (7.8)** | **0.127** | **.000 (0.3)** | **.180 (72.0)** |
|  | **Frontal, caudal middle** | **Left** | **-.11 (46.2)** | **0.102** | **.000 (0.1)** | **.000 (0.3)** | **.036 (-12.5)** | **.041 (-14.3)** | **-.04 (15.0)** | **0.123** | **.000 (0.4)** | **.117 (47.1)** |
|  |  | **Right** | **-.10 (39.3)** | **0.096** | **.000 (-0.1)** | **.000 (-0.0)** | **.038 (-12.8)** | **.048 (-16.1)** | **-.02 (4.1)** | **0.120** | **.000 (0.0)** | **.141 (54.4)** |
|  | **Frontal, rostral middle** | **Left** | **-.10 (42.1)** | **0.108** | **.000 (0.2)** | **.000 (0.3)** | **.032 (-11.3)** | **.041 (-14.1)** | **-.06 (18.6)** | **0.150** | **.000 (0.5)** | **.150 (60.5)** |
|  |  | **Right** | **-.11 (45.3)** | **0.110** | **.000 (0.2)** | **.000 (0.0)** | **.033 (-11.0)** | **.039 (-13.1)** | **-.04 (6.6)** | **0.146** | **.000 (0.1)** | **.175 (68.6)** |
|  | **Frontal, pars opercularis** | **Left** | **-.08 (33.0)** | **0.098** | **.000 (-0.1)** | **.000 (-0.0)** | **.016 (-5.9)** | **.045 (-15.5)** | **-.05 (19.5)** | **0.128** | **.001 (0.9)** | **.098 (41.7)** |
|  |  | **Right** | **-.05 (13.3)** | **0.084** | **.000 (0.3)** | **.000 (0.2)** | **.021 (-7.3)** | **.041 (-13.6)** | **-.02 (3.4)** | **--** | **.000 (0.0)** | **.122 (50.9)** |
|  | **Frontal, pars triangularis** | **Left** | **-.07 (18.2)** | **0.096** | **.000 (-0.2)** | **.000 (-0.3)** | **.002 (-1.0)** | **.007 (-2.8)** | **-.05 (14.9)** | **0.133** | **.001 (0.7)** | **.100 (40.1)** |
|  |  | **Right** | **-.07 (18.0)** | **0.090** | **.000 (0.1)** | **.000 (0.2)** | **.008 (-3.0)** | **.014 (-5.0)** | **-.04 (7.0)** | **0.144** | **.000 (0.2)** | **.104 (40.8)** |
|  | **Frontal, pars orbitalis** | **Left** | **-.08 (26.7)** | **0.088** | **.001 (0.6)** | **.001 (0.4)** | **.011 (-4.1)** | **.023 (-8.3)** | **-.05 (19.5)** | **0.132** | **.001 (0.7)** | **.107 (43.6)** |
|  |  | **Right** | **-.07 (23.7)** | **0.089** | **.000 (0.0)** | **.000 (0.1)** | **.021 (-7.3)** | **.030 (-10.1)** | **-.02 (4.3)** | **0.131** | **.000 (0.1)** | **.127 (51.7)** |
|  | **Frontal, lateral orbital** | **Left** | **-.08 (25.9)** | **0.085** | **.000 (0.2)** | **.000 (0.1)** | **.001 (-0.8)** | **.005 (-2.2)** | **-.06 (22.6)** | **0.140** | **.001 (1.1)** | **.087 (36.6)** |
|  |  | **Right** | **-.06 (10.9)** | **0.079** | **.000 (0.0)** | **.000 (0.2)** | **.000 (0.0)** | **.001 (0.8)** | **-.03 (5.9)** | **0.135** | **.000 (-0.1)** | **.102 (-39.1)** |
|  | **Frontal, medial orbital** | **Left** | **-.07 (15.8)** | **0.078** | **.000 (-0.3)** | **.000 (-0.1)** | **.012 (-4.3)** | **.019 (-6.8)** | **-.04 (10.0)** | **0.129** | **.000 (0.4)** | **.086 (32.9)** |
|  |  | **Right** | **-.08 (26.9)** | **0.083** | **.000 (0.1)** | **.000 (0.1)** | **.007 (-2.8)** | **.018 (-6.2)** | **-.04 (5.7)** | **0.117** | **.000 (0.1)** | **.078 (28.8)** |
|  | **Frontal, pole** | **Left** | **-.10 (30.8)** | **0.095** | **.001 (0.5)** | **.000 (0.2)** | **.015 (-5.2)** | **.017 (-6.1)** | **-.07 (15.1)** | **0.153** | **.000 (0.4)** | **.094 (34.8)** |
|  |  | **Right** | **-.10 (30.9)** | **0.094** | **.000 (0.3)** | **.000 (0.1)** | **.009 (-3.3)** | **.016 (-5.8)** | **-.05 (9.1)** | **0.140** | **.000 (-0.1)** | **.095 (-35.1)** |
|  | **Frontal, precentral gyrus** | **Left** | **-.10 (42.6)** | **0.072** | **.000 (0.0)** | **.001 (0.4)** | **.017 (-6.1)** | **.027 (-9.3)** | **-.04 (16.1)** | **0.103** | **.000 (0.3)** | **.063 (25.4)** |
|  |  | **Right** | **-.06 (19.8)** | **0.064** | **.000 (0.1)** | **.000 (0.3)** | **.025 (-8.6)** | **.046 (-15.7)** | **-.01 (1.4)** | **--** | **.000 (0.2)** | **.075 (29.2)** |
|  | **Parietal, postcentral gyrus** | **Left** | **-.09 (30.2)** | **0.081** | **.000 (-0.1)** | **.000 (-0.2)** | **.005 (-2.1)** | **.023 (-8.1)** | **-.04 (12.6)** | **0.114** | **.001 (0.8)** | **.034 (14.2)** |
|  |  | **Right** | **-.03 (4.6)** | **0.060** | **.001 (-0.5)** | **.000 (-0.4)** | **.009 (-3.3)** | **.031 (-10.5)** | **-.01 (0.8)** | **--** | **.000 (0.1)** | **.039 (15.3)** |
|  | **Parietal, paracentral gyrus** | **Left** | **-.09 (35.1)** | **0.076** | **.000 (-0.3)** | **.000 (-0.2)** | **.035 (-11.9)** | **.040 (-13.5)** | **-.04 (14.8)** | **0.100** | **.000 (0.3)** | **.053 (21.1)** |
|  |  | **Right** | **-.09 (29.9)** | **0.070** | **.000 (0.0)** | **.000 (0.2)** | **.022 (-7.8)** | **.036 (-12.5)** | **-.03 (8.4)** | **0.104** | **.000 (0.4)** | **.088 (34.2)** |
|  | **Parietal, superior** | **Left** | **-.10 (40.2)** | **0.105** | **.000 (-0.2)** | **.000 (-0.1)** | **.024 (-8.4)** | **.037 (-12.8)** | **-.05 (20.6)** | **0.130** | **.000 (0.4)** | **.101 (42.2)** |
|  |  | **Right** | **-.05 (9.7)** | **0.088** | **.000 (-0.3)** | **.000 (-0.4)** | **.012 (-4.6)** | **.028 (-9.8)** | **-.04 (9.2)** | **0.124** | **.000 (0.1)** | **.092 (36.3)** |
|  | **Parietal, inferior** | **Left** | **-.07 (23.0)** | **0.098** | **.000 (0.1)** | **.000 (0.0)** | **.011 (-4.2)** | **.022 (-7.7)** | **-.06 (24.5)** | **0.137** | **.001 (0.6)** | **.078 (33.1)** |
|  |  | **Right** | **-.02 (3.5)** | **--** | **.000 (-0.4)** | **.000 (-0.3)** | **.003 (-1.4)** | **.003 (-1.4)** | **-.04 (10.9)** | **0.123** | **.000 (-0.2)** | **.089 (-37.2)** |
|  | **Parietal, supramarginal** | **Left** | **-.09 (32.6)** | **0.103** | **.000 (-0.2)** | **.000 (-0.3)** | **.014 (-5.1)** | **.020 (-6.9)** | **-.05 (19.8)** | **0.132** | **.001 (0.7)** | **.086 (37.1)** |
|  |  | **Right** | **-.01 (0.4)** | **--** | **.000 (0.1)** | **.000 (0.2)** | **.021 (-7.3)** | **.023 (-8.2)** | **-.02 (3.3)** | **--** | **.000 (-0.3)** | **.097 (-41.1)** |
|  | **Parietal, precuneus** | **Left** | **-.07 (26.7)** | **0.095** | **.001 (-0.5)** | **.001 (-0.5)** | **.018 (-6.4)** | **.041 (-14.0)** | **-.05 (20.4)** | **0.131** | **.000 (0.3)** | **.096 (40.9)** |
|  |  | **Right** | **-.04 (8.5)** | **0.081** | **.000 (-0.2)** | **.001 (-0.4)** | **.012 (-4.7)** | **.030 (-10.6)** | **-.04 (14.7)** | **0.125** | **.000 (0.2)** | **.097 (41.1)** |
|  | **Temporal, parahippocampal** | **Left** | **-.00 (0.1)** | **--** | **.000 (-0.4)** | **.001 (-0.6)** | **.001 (0.8)** | **.007 (2.6)** | **-.04 (7.4)** | **0.143** | **.001 (0.7)** | **.023 (9.9)** |
|  |  | **Right** | **-.06 (11.1)** | **0.062** | **.000 (-0.2)** | **.001 (-0.5)** | **.002 (0.9)** | **.008 (3.0)** | **-.01 (1.1)** | **--** | **.000 (0.2)** | **.014 (6.2)** |
|  | **Temporal, entorhinal** | **Left** | **-.01 (0.6)** | **--** | **.004 (-1.8)** | **.004 (-1.7)** | **.001 (0.6)** | **.000 (0.2)** | **-.05 (11.5)** | **0.116** | **.002 (1.1)** | **.010 (4.4)** |
|  |  | **Right** | **-.01 (1.0)** | **--** | **.002 (-1.2)** | **.003 (-1.6)** | **.000 (0.1)** | **.002 (1.2)** | **-.02 (2.6)** | **--** | **.001 (0.9)** | **.034 (13.5)** |
|  | **Temporal, pole** | **Left** | **-.03 (2.4)** | **--** | **.000 (0.1)** | **.000 (0.1)** | **.002 (-1.0)** | **.006 (-2.4)** | **-.04 (4.6)** | **0.146** | **.000 (0.5)** | **.035 (13.6)** |
|  |  | **Right** | **-.01 (0.6)** | **--** | **.001 (-0.8)** | **.002 (-1.0)** | **.000 (0.0)** | **.000 (0.1)** | **-.04 (7.9)** | **0.114** | **.000 (0.0)** | **.031 (12.3)** |
|  | **Temporal, superior** | **Left** | **-.05 (10.8)** | **0.093** | **.000 (0.0)** | **.000 (0.0)** | **.005 (-2.1)** | **.016 (-5.7)** | **-.05 (20.4)** | **0.140** | **.000 (0.4)** | **.070 (32.4)** |
|  |  | **Right** | **-.03 (4.5)** | **0.065** | **.001 (-0.5)** | **.001 (-0.7)** | **.009 (-3.4)** | **.029 (-10.0)** | **-.01 (1.1)** | **--** | **.000 (0.0)** | **.075 (34.6)** |
|  | **Temporal, middle** | **Left** | **-.03 (4.6)** | **0.089** | **.000 (0.2)** | **.000 (0.0)** | **.002 (-1.2)** | **.012 (-4.3)** | **-.06 (26.3)** | **0.136** | **.000 (0.2)** | **.064 (30.3)** |
|  |  | **Right** | **-.07 (22.4)** | **0.053** | **.001 (-0.7)** | **.002 (-0.9)** | **.001 (-0.7)** | **.011 (-4.2)** | **-.01 (0.9)** | **--** | **.000 (-0.4)** | **.079 (-36.7)** |
|  | **Temporal, inferior** | **Left** | **-.00 (0.2)** | **--** | **.000 (0.0)** | **.000 (0.2)** | **.000 (0.0)** | **.008 (3.0)** | **-.06 (23.9)** | **0.140** | **.000 (0.5)** | **.060 (28.1)** |
|  |  | **Right** | **-.07 (26.5)** | **0.041** | **.003 (-1.2)** | **.006 (-2.5)** | **.000 (0.0)** | **.004 (1.7)** | **-.02 (2.3)** | **--** | **.000 (-0.2)** | **.054 (-25.0)** |
|  | **Temporal, transverse** | **Left** | **-.02 (2.2)** | **--** | **.000 (0.0)** | **.000 (0.1)** | **.000 (-0.1)** | **.004 (-1.9)** | **-.05 (16.4)** | **0.108** | **.000 (0.5)** | **.044 (17.7)** |
|  |  | **Right** | **-.04 (6.7)** | **0.029** | **.000 (0.0)** | **.000 (0.3)** | **.003 (-1.3)** | **.007 (-2.6)** | **-.02 (4.7)** | **0.091** | **.000 (0.0)** | **.045 (17.9)** |
|  | **Temporal, bank sup temp sulc** | **Left** | **-.01 (1.1)** | **--** | **.000 (0.1)** | **.000 (0.0)** | **.003 (-1.3)** | **.006 (-2.5)** | **-.05 (15.0)** | **0.132** | **.000 (0.3)** | **.047 (20.7)** |
|  |  | **Right** | **-.07 (24.7)** | **0.047** | **.000 (-0.1)** | **.000 (-0.0)** | **.002 (-0.9)** | **.003 (-1.5)** | **-.01 (0.5)** | **--** | **.000 (-0.1)** | **.055 (-23.8)** |
|  | **Temporal, fusiform** | **Left** | **-.00 (0.0)** | **--** | **.000 (-0.1)** | **.001 (-0.7)** | **.000 (0.0)** | **.007 (2.7)** | **-.06 (24.7)** | **0.134** | **.000 (0.4)** | **.055 (25.9)** |
|  |  | **Right** | **-.06 (24.1)** | **0.036** | **.001 (-0.5)** | **.003 (-1.5)** | **.000 (0.4)** | **.001 (0.8)** | **-.03 (5.8)** | **0.117** | **.000 (0.1)** | **.060 (29.6)** |
|  | **Occipital, lateral** | **Left** | **-.02 (1.9)** | **--** | **.000 (0.2)** | **.000 (0.1)** | **.001 (0.5)** | **.003 (1.5)** | **-.06 (16.2)** | **0.141** | **.000 (0.3)** | **.034 (15.0)** |
|  |  | **Right** | **-.12 (51.5)** | **0.025** | **.000 (-0.1)** | **.001 (-0.7)** | **.004 (1.7)** | **.001 (0.7)** | **-.02 (3.1)** | **--** | **.000 (-0.3)** | **.059 (-25.7)** |
|  | **Occipital, pericalcarine** | **Left** | **-.02 (2.5)** | **--** | **.003 (-1.3)** | **.001 (-0.5)** | **.008 (2.9)** | **.000 (0.1)** | **-.03 (3.0)** | **--** | **.000 (0.3)** | **.006 (2.6)** |
|  |  | **Right** | **-.06 (13.7)** | **0.016** | **.000 (-0.4)** | **.001 (-0.5)** | **.018 (6.3)** | **.003 (1.5)** | **-.00 (0.1)** | **--** | **.000 (0.2)** | **.011 (4.6)** |
|  | **Occipital, lingual** | **Left** | **-.04 (9.0)** | **0.034** | **.001 (-0.5)** | **.001 (-0.4)** | **.001 (0.5)** | **.000 (0.0)** | **-.04 (6.0)** | **0.135** | **.001 (0.7)** | **.022 (9.6)** |
|  |  | **Right** | **-.08 (26.1)** | **0.036** | **.000 (-0.3)** | **.004 (-1.7)** | **.014 (5.1)** | **.006 (2.6)** | **-.01 (0.9)** | **--** | **.000 (0.0)** | **.031 (13.2)** |
|  | **Occipital, cuneus** | **Left** | **-.01 (0.5)** | **--** | **.000 (0.3)** | **.000 (0.1)** | **.001 (0.8)** | **.000 (0.4)** | **-.03 (4.9)** | **0.144** | **.001 (0.7)** | **.050 (20.4)** |
|  |  | **Right** | **-.05 (11.5)** | **0.037** | **.000 (0.1)** | **.001 (0.6)** | **.011 (4.1)** | **.000 (0.3)** | **-.02 (1.6)** | **--** | **.000 (0.0)** | **.059 (23.8)** |
| **Volume (cortical)** | **Cingulate, caudal ant** | **Left** | **-.02 (2.5)** | **--** | **.002 (1.2)** | **.000 (0.3)** | **.000 (-0.4)** | **.001 (-0.8)** | **-.02 (33.9)** | **0.000** | **.000 (-0.1)** | **.015 (-5.6)** |
|  |  | **Right** | **-.02 (2.9)** | **--** | **.000 (0.0)** | **.002 (0.9)** | **.001 (-0.7)** | **.002 (-1.2)** | **-.02 (32.2)** | **-0.005** | **.002 (-1.4)** | **.006 (-2.7)** |
|  | **Cingulate, rostral ant** | **Left** | **-.03 (7.5)** | **0.050** | **.001 (-0.5)** | **.002 (-0.9)** | **.001 (-0.6)** | **.003 (-1.5)** | **-.03 (32.3)** | **0.001** | **.002 (0.9)** | **.018 (6.8)** |
|  |  | **Right** | **-.02 (7.4)** | **0.032** | **.000 (0.3)** | **.001 (0.5)** | **.007 (-2.8)** | **.002 (-0.9)** | **-.02 (20.4)** | **-0.006** | **.000 (-0.4)** | **.002 (-1.3)** |
|  | **Cingulate, posterior** | **Left** | **-.03 (9.3)** | **0.062** | **.000 (-0.3)** | **.003 (-1.2)** | **.001 (0.5)** | **.001 (0.5)** | **-.04 (59.2)** | **0.001** | **.000 (-0.1)** | **.021 (-7.9)** |
|  |  | **Right** | **-.03 (5.7)** | **0.027** | **.000 (-0.1)** | **.000 (-0.4)** | **.006 (-2.4)** | **.003 (-1.3)** | **-.03 (47.7)** | **-0.002** | **.000 (-0.2)** | **.003 (-1.3)** |
|  | **Cingulate, isthmus** | **Left** | **-.02 (6.8)** | **0.038** | **.000 (0.1)** | **.000 (0.4)** | **.001 (-0.5)** | **.000 (-0.3)** | **-.03 (40.4)** | **0.004** | **.000 (-0.2)** | **.017 (-6.2)** |
|  |  | **Right** | **-.02 (6.1)** | **0.039** | **.000 (-0.3)** | **.005 (-1.9)** | **.001 (0.7)** | **.003 (1.5)** | **-.03 (30.7)** | **0.009** | **.000 (0.0)** | **.006 (2.7)** |
|  | **Insula** | **Left** | **-.02 (3.4)** | **--** | **.000 (-0.2)** | **.001 (-0.6)** | **.003 (-1.3)** | **.001 (-0.8)** | **-.03 (29.0)** | **0.000** | **.000 (-0.4)** | **.003 (-1.2)** |
|  |  | **Right** | **-.02 (3.0)** | **--** | **.000 (-0.1)** | **.000 (-0.2)** | **.000 (0.0)** | **.000 (0.2)** | **-.03 (25.3)** | **0.007** | **.002 (-0.9)** | **.009 (-3.6)** |
|  | **Frontal, superior** | **Left** | **-.03 (12.4)** | **0.065** | **.000 (0.1)** | **.001 (0.5)** | **.002 (-1.1)** | **.009 (-3.3)** | **-.04 (38.9)** | **-0.015** | **.000 (-0.1)** | **.012 (-4.6)** |
|  |  | **Right** | **-.03 (8.0)** | **0.055** | **.000 (0.3)** | **.003 (1.6)** | **.000 (-0.1)** | **.002 (-1.0)** | **-.03 (32.6)** | **-0.015** | **.000 (-0.4)** | **.010 (-4.0)** |
|  | **Frontal, caudal middle** | **Left** | **-.01 (2.8)** | **--** | **.001 (0.7)** | **.000 (0.0)** | **.000 (-0.3)** | **.002 (-1.0)** | **-.03 (30.5)** | **-0.009** | **.000 (-0.5)** | **.009 (-3.4)** |
|  |  | **Right** | **-.02 (3.5)** | **--** | **.000 (0.3)** | **.002 (1.1)** | **.000 (-0.1)** | **.000 (-0.2)** | **-.03 (23.9)** | **-0.012** | **.000 (-0.5)** | **.006 (-2.6)** |
|  | **Frontal, rostral middle** | **Left** | **-.04 (20.1)** | **0.061** | **.000 (0.2)** | **.001 (0.8)** | **.002 (-1.1)** | **.016 (-5.7)** | **-.04 (50.5)** | **-0.003** | **.000 (-0.5)** | **.031 (-11.2)** |
|  |  | **Right** | **-.03 (11.8)** | **0.049** | **.001 (0.4)** | **.004 (1.7)** | **.002 (-1.1)** | **.008 (-3.0)** | **-.03 (45.5)** | **-0.004** | **.000 (0.0)** | **.033 (12.2)** |
|  | **Frontal, pars opercularis** | **Left** | **-.03 (11.5)** | **0.052** | **.001 (0.5)** | **.000 (0.2)** | **.002 (-0.8)** | **.009 (-3.2)** | **-.03 (47.1)** | **-0.003** | **.002 (-1.1)** | **.011 (-4.4)** |
|  |  | **Right** | **-.03 (8.4)** | **0.037** | **.001 (0.7)** | **.001 (0.6)** | **.000 (-0.1)** | **.000 (-0.0)** | **-.03 (43.1)** | **-0.003** | **.000 (-0.5)** | **.017 (-6.5)** |
|  | **Frontal, pars triangularis** | **Left** | **-.05 (15.3)** | **0.050** | **.000 (-0.1)** | **.002 (-0.9)** | **.001 (-0.7)** | **.010 (-3.8)** | **-.04 (33.9)** | **-0.006** | **.000 (-0.2)** | **.009 (-3.8)** |
|  |  | **Right** | **-.03 (5.1)** | **0.039** | **.001 (0.7)** | **.002 (0.9)** | **.000 (-0.3)** | **.003 (-1.5)** | **-.04 (48.9)** | **0.001** | **.000 (0.3)** | **.017 (6.3)** |
|  | **Frontal, pars orbitalis** | **Left** | **-.04 (19.2)** | **0.069** | **.000 (0.0)** | **.001 (0.6)** | **.000 (-0.4)** | **.006 (-2.3)** | **-.03 (44.4)** | **-0.006** | **.000 (-0.2)** | **.013 (-5.1)** |
|  |  | **Right** | **-.03 (9.0)** | **0.055** | **.001 (0.6)** | **.000 (0.3)** | **.001 (-0.7)** | **.007 (-2.7)** | **-.03 (54.9)** | **0.000** | **.000 (-0.4)** | **.023 (-8.7)** |
|  | **Frontal, lateral orbital** | **Left** | **-.04 (15.6)** | **0.066** | **.000 (-0.1)** | **.002 (-1.0)** | **.002 (-0.9)** | **.002 (-1.0)** | **-.04 (44.8)** | **0.012** | **.000 (0.2)** | **.058 (20.7)** |
|  |  | **Right** | **-.02 (2.7)** | **--** | **.000 (0.1)** | **.003 (1.3)** | **.000 (-0.3)** | **.000 (-0.3)** | **-.03 (16.2)** | **0.008** | **.000 (-0.4)** | **.022 (-8.2)** |
|  | **Frontal, medial orbital** | **Left** | **-.05 (14.4)** | **0.075** | **.000 (0.1)** | **.002 (0.9)** | **.000 (0.2)** | **.000 (0.2)** | **-.04 (28.8)** | **0.015** | **.000 (-0.1)** | **.013 (-5.0)** |
|  |  | **Right** | **-.05 (12.9)** | **0.061** | **.000 (-0.3)** | **.006 (-2.2)** | **.001 (0.5)** | **.000 (0.0)** | **-.03 (14.6)** | **0.013** | **.002 (-1.1)** | **.043 (-15.2)** |
|  | **Frontal, pole** | **Left** | **-.04 (5.2)** | **0.067** | **.000 (0.0)** | **.002 (0.9)** | **.000 (0.0)** | **.001 (0.5)** | **-.06 (41.2)** | **-0.001** | **.000 (0.2)** | **.012 (5.1)** |
|  |  | **Right** | **-.03 (2.7)** | **--** | **.000 (-0.1)** | **.002 (-1.1)** | **.002 (1.2)** | **.001 (0.7)** | **-.05 (28.3)** | **0.000** | **.000 (0.3)** | **.012 (5.0)** |
|  | **Frontal, precentral gyrus** | **Left** | **-.03 (7.5)** | **0.078** | **.000 (0.0)** | **.001 (0.5)** | **.010 (-3.7)** | **.008 (-3.1)** | **-.04 (25.1)** | **-0.007** | **.001 (-0.9)** | **.009 (-3.6)** |
|  |  | **Right** | **-.03 (6.4)** | **0.073** | **.000 (0.4)** | **.002 (0.8)** | **.003 (-1.4)** | **.001 (-0.6)** | **-.04 (24.1)** | **-0.004** | **.002 (-1.0)** | **.010 (-4.2)** |
|  | **Parietal, postcentral gyrus** | **Left** | **-.03 (8.0)** | **0.069** | **.000 (0.1)** | **.000 (0.3)** | **.008 (-3.1)** | **.005 (-2.1)** | **-.03 (27.0)** | **-0.004** | **.000 (-0.4)** | **.008 (-3.3)** |
|  |  | **Right** | **-.03 (5.2)** | **0.065** | **.001 (0.7)** | **.001 (0.7)** | **.001 (-0.6)** | **.001 (-0.7)** | **-.04 (30.1)** | **-0.003** | **.001 (-0.5)** | **.017 (-6.2)** |
|  | **Parietal, paracentral gyrus** | **Left** | **-.03 (8.1)** | **0.077** | **.000 (0.2)** | **.000 (0.2)** | **.008 (-3.0)** | **.002 (-1.2)** | **-.04 (27.2)** | **-0.005** | **.000 (-0.3)** | **.021 (-7.9)** |
|  |  | **Right** | **-.03 (8.2)** | **0.058** | **.000 (0.0)** | **.001 (0.7)** | **.002 (-1.1)** | **.000 (-0.0)** | **-.03 (16.9)** | **-0.016** | **.001 (-0.5)** | **.015 (-5.8)** |
|  | **Parietal, superior** | **Left** | **-.03 (9.6)** | **0.072** | **.000 (-0.2)** | **.002 (-1.0)** | **.002 (-0.9)** | **.005 (-2.0)** | **-.04 (37.3)** | **-0.002** | **.000 (-0.4)** | **.020 (-7.6)** |
|  |  | **Right** | **-.03 (7.9)** | **0.064** | **.000 (0.0)** | **.003 (1.5)** | **.000 (-0.2)** | **.001 (-0.8)** | **-.04 (37.3)** | **-0.003** | **.001 (-0.5)** | **.010 (-3.9)** |
|  | **Parietal,**  **inferior** | **Left** | **-.04 (25.5)** | **0.063** | **.000 (0.1)** | **.002 (1.0)** | **.002 (-1.2)** | **.010 (-3.8)** | **-.04 (60.4)** | **0.004** | **.000 (0.1)** | **.026 (9.4)** |
|  |  | **Right** | **-.04 (22.8)** | **0.050** | **.000 (0.1)** | **.002 (0.9)** | **.000 (-0.1)** | **.002 (-0.9)** | **-.04 (72.0)** | **0.005** | **.000 (-0.4)** | **.014 (-5.4)** |
|  | **Parietal,**  **supramarginal** | **Left** | **-.03 (14.0)** | **0.052** | **.001 (-0.5)** | **.004 (-1.7)** | **.001 (-0.8)** | **.007 (-2.6)** | **-.03 (54.3)** | **0.002** | **.000 (0.0)** | **.024 (8.9)** |
|  |  | **Right** | **-.03 (9.3)** | **0.044** | **.000 (0.1)** | **.001 (0.5)** | **.000 (0.1)** | **.001 (0.4)** | **-.04 (58.8)** | **0.003** | **.000 (-0.1)** | **.018 (-6.6)** |
|  | **Parietal, precuneus** | **Left** | **-.04 (15.9)** | **0.067** | **.000 (0.1)** | **.002 (1.0)** | **.000 (-0.4)** | **.005 (-2.1)** | **-.04 (62.4)** | **0.006** | **.000 (0.0)** | **.017 (6.2)** |
|  |  | **Right** | **-.03 (11.4)** | **0.057** | **.001 (0.8)** | **.001 (0.8)** | **.000 (0.0)** | **.000 (0.3)** | **-.04 (55.8)** | **0.004** | **.000 (-0.4)** | **.015 (-5.7)** |
|  | **Temporal, parahippocampal** | **Left** | **-.04 (8.5)** | **0.070** | **.001 (-0.5)** | **.001 (-0.5)** | **.002 (-1.0)** | **.000 (-0.2)** | **-.03 (28.3)** | **0.014** | **.000 (-0.3)** | **.007 (-2.8)** |
|  |  | **Right** | **-.03 (4.5)** | **0.056** | **.000 (0.1)** | **.001 (0.5)** | **.000 (0.2)** | **.001 (0.5)** | **-.03 (23.0)** | **0.017** | **.001 (-0.8)** | **.048 (-17.0)** |
|  | **Temporal, entorhinal** | **Left** | **-.03 (2.9)** | **--** | **.000 (-0.1)** | **.000 (-0.1)** | **.001 (0.6)** | **.000 (0.2)** | **-.03 (13.1)** | **0.009** | **.000 (0.0)** | **.009 (3.7)** |
|  |  | **Right** | **-.01 (1.1)** | **--** | **.002 (0.8)** | **.000 (0.2)** | **.001 (0.4)** | **.002 (0.8)** | **-.02 (7.4)** | **0.009** | **.001 (-0.6)** | **.000 (-0.1)** |
|  | **Temporal, pole** | **Left** | **--** | **--** | **--** | **--** | **--** | **--** | **-.04 (12.4)** | **0.014** | **.001 (0.6)** | **.009 (3.6)** |
|  |  | **Right** | **--** | **--** | **--** | **--** | **--** | **--** | **-.05 (28.8)** | **0.015** | **.001 (-0.7)** | **.013 (-5.2)** |
|  | **Temporal, superior** | **Left** | **-.04 (20.3)** | **0.068** | **.000 (0.4)** | **.000 (0.3)** | **.004 (-1.8)** | **.007 (-2.8)** | **-.04 (96.9)** | **0.007** | **.000 (0.0)** | **.030 (11.1)** |
|  |  | **Right** | **-.05 (28.6)** | **0.053** | **.000 (0.1)** | **.003 (1.2)** | **.001 (-0.6)** | **.002 (-1.2)** | **-.04 (103.7)** | **0.008** | **.001 (-0.8)** | **.030 (-10.9)** |
|  | **Temporal, middle** | **Left** | **-.06 (35.6)** | **0.070** | **.000 (0.2)** | **.001 (0.5)** | **.003 (-1.2)** | **.011 (-4.1)** | **-.04 (108.2)** | **0.014** | **.001 (0.5)** | **.040 (14.1)** |
|  |  | **Right** | **-.04 (22.6)** | **0.057** | **.000 (0.3)** | **.003 (1.4)** | **.001 (-0.7)** | **.007 (-2.6)** | **-.05 (127.8)** | **0.014** | **.000 (0.0)** | **.042 (15.3)** |
|  | **Temporal, inferior** | **Left** | **-.05 (29.2)** | **0.054** | **.001 (0.7)** | **.000 (0.0)** | **.003 (-1.5)** | **.008 (-3.2)** | **-.04 (69.3)** | **0.019** | **.001 (0.4)** | **.048 (17.2)** |
|  |  | **Right** | **-.03 (16.4)** | **0.043** | **.001 (0.8)** | **.000 (0.3)** | **.000 (-0.2)** | **.003 (-1.3)** | **-.04 (84.5)** | **0.019** | **.000 (0.0)** | **.046 (16.2)** |
|  | **Temporal, transverse** | **Left** | **-.02 (3.8)** | **--** | **.000 (0.3)** | **.000 (0.3)** | **.000 (-0.1)** | **.000 (-0.1)** | **-.04 (42.3)** | **0.002** | **.000 (-0.2)** | **.001 (-0.9)** |
|  |  | **Right** | **-.02 (4.6)** | **0.038** | **.001 (-0.7)** | **.004 (-1.8)** | **.001 (-0.4)** | **.000 (-0.1)** | **-.03 (24.4)** | **0.012** | **.000 (0.2)** | **.010 (4.1)** |
|  | **Temporal, bank sup temp sulc** | **Left** | **-.04 (20.0)** | **0.036** | **.000 (0.0)** | **.001 (0.6)** | **.002 (-0.9)** | **.001 (-0.5)** | **-.02 (28.8)** | **0.006** | **.000 (-0.3)** | **.014 (-5.4)** |
|  |  | **Right** | **-.04 (15.4)** | **0.023** | **.000 (-0.1)** | **.000 (-0.3)** | **.002 (-1.1)** | **.001 (-0.7)** | **-.04 (48.3)** | **0.008** | **.000 (-0.4)** | **.006 (-2.8)** |
|  | **Temporal, fusiform** | **Left** | **-.05 (27.9)** | **0.060** | **.000 (0.0)** | **.000 (0.3)** | **.002 (-1.1)** | **.003 (-1.4)** | **-.04 (66.1)** | **0.023** | **.000 (-0.2)** | **.044 (-15.9)** |
|  |  | **Right** | **-.03 (11.6)** | **0.055** | **.002 (0.8)** | **.000 (0.3)** | **.000 (0.0)** | **.001 (0.5)** | **-.04 (68.4)** | **0.021** | **.001 (-0.4)** | **.026 (-9.6)** |
|  | **Occipital, lateral** | **Left** | **-.04 (14.8)** | **0.078** | **.000 (0.3)** | **.000 (0.4)** | **.000 (-0.4)** | **.004 (-1.8)** | **-.05 (87.0)** | **0.021** | **.000 (-0.1)** | **.017 (-6.3)** |
|  |  | **Right** | **-.03 (7.1)** | **0.049** | **.001 (0.7)** | **.002 (0.8)** | **.000 (-0.3)** | **.000 (-0.2)** | **-.05 (78.1)** | **0.022** | **.001 (-0.5)** | **.029 (-10.6)** |
|  | **Occipital, pericalcarine** | **Left** | **-.00 (0.4)** | **--** | **.005 (2.1)** | **.000 (0.2)** | **.000 (0.0)** | **.000 (0.0)** | **-.02 (5.5)** | **0.011** | **.000 (-0.2)** | **.001 (-0.7)** |
|  |  | **Right** | **-.01 (2.3)** | **--** | **.006 (2.3)** | **.000 (0.0)** | **.002 (1.2)** | **.004 (1.9)** | **-.02 (8.5)** | **0.010** | **.000 (0.1)** | **.005 (2.3)** |
|  | **Occipital, lingual** | **Left** | **-.02 (3.7)** | **--** | **.001 (0.5)** | **.000 (0.1)** | **.003 (-1.4)** | **.000 (-0.2)** | **-.04 (61.3)** | **0.016** | **.000 (-0.3)** | **.010 (-4.1)** |
|  |  | **Right** | **-.02 (2.5)** | **--** | **.002 (0.8)** | **.001 (0.6)** | **.000 (0.3)** | **.001 (0.6)** | **-.04 (51.3)** | **0.017** | **.000 (-0.2)** | **.007 (-2.9)** |
|  | **Occipital, cuneus** | **Left** | **-.02 (5.0)** | **0.062** | **.001 (0.4)** | **.000 (0.3)** | **.000 (-0.2)** | **.000 (-0.1)** | **-.03 (31.8)** | **0.010** | **.000 (0.2)** | **.006 (2.5)** |
|  |  | **Right** | **-.02 (4.2)** | **0.049** | **.001 (0.5)** | **.001 (0.6)** | **.002 (1.2)** | **.002 (0.8)** | **-.03 (24.2)** | **0.010** | **.001 (-0.6)** | **.005 (-2.3)** |
| **Volume (subc.)** | **3rd Ventricle** | **Bil** | **.06 (67.9)** | **-0.019** | **.001 (0.4)** | **.000 (0.2)** | **.001 (0.6)** | **.003 (1.4)** | **-.05 (123.5)** | **-0.004** | **.008 (3.4)** | **.002 (1.2)** |
|  | **4th Ventricle** | **Bil** | **.02 (11.4)** | **0.000** | **.001 (0.4)** | **.001 (0.5)** | **.011 (4.1)** | **.004 (1.8)** | **-.02 (17.6)** | **0.015** | **.008 (3.2)** | **.007 (2.8)** |
|  | **5th Ventricle** | **Bil** | **.00 (0.1)** | **--** | **.000 (0.0)** | **.000 (0.1)** | **.001 (-0.6)** | **.000 (-0.3)** | **-.05 (3.4)** | **--** | **.002 (-0.9)** | **.005 (-2.1)** |
|  | **Inf lat vent** | **Left** | **.05 (38.3)** | **-0.002** | **.007 (3.0)** | **.001 (0.7)** | **.013 (4.9)** | **.006 (2.4)** | **-.06 (71.1)** | **0.002** | **.024 (9.3)** | **.009 (5.0)** |
|  |  | **Right** | **.05 (44.0)** | **-0.008** | **.031 (11.2)** | **.018 (6.7)** | **.011 (4.2)** | **.009 (3.6)** | **-.06 (62.8)** | **0.002** | **.020 (7.6)** | **.005 (2.7)** |
|  | **Lat vent** | **Left** | **.05 (121.2)** | **-0.005** | **.032 (12.2)** | **.016 (6.4)** | **.013 (5.2)** | **.007 (3.0)** | **-.05 (171.2)** | **-0.008** | **.025 (10.9)** | **.012 (6.2)** |
|  |  | **Right** | **.05 (123.9)** | **-0.005** | **.031 (11.7)** | **.015 (6.0)** | **.010 (4.1)** | **.005 (2.3)** | **-.05 (182.8)** | **-0.009** | **.026 (11.7)** | **.011 (6.2)** |
|  | **CSF total** | **Bil** | **-.03 (8.1)** | **-0.046** | **.001 (-0.8)** | **.001 (-0.4)** | **.010 (-3.7)** | **.001 (-0.8)** | **-.03 (17.3)** | **-0.009** | **.010 (3.6)** | **.003 (1.5)** |
|  | **Accumbens** | **Left** | **-.08 (26.7)** | **0.038** | **.000 (0.4)** | **.000 (0.3)** | **.003 (-1.3)** | **.005 (-2.0)** | **-.04 (9.4)** | **0.033** | **.002 (-1.0)** | **.017 (-6.4)** |
|  |  | **Right** | **-.05 (16.7)** | **0.034** | **.000 (-0.3)** | **.001 (-0.8)** | **.003 (-1.5)** | **.000 (-0.0)** | **-.02 (5.7)** | **-0.003** | **.000 (0.0)** | **.007 (2.9)** |
|  | **Amygdala** | **Left** | **-.05 (15.3)** | **0.030** | **.000 (-0.3)** | **.001 (-0.6)** | **.008 (-3.1)** | **.003 (-1.3)** | **-.03 (13.5)** | **0.007** | **.000 (0.3)** | **.006 (2.6)** |
|  |  | **Right** | **-.04 (11.5)** | **0.011** | **.000 (0.4)** | **.000 (0.4)** | **.002 (-1.1)** | **.000 (-0.1)** | **-.03 (15.4)** | **0.003** | **.003 (-1.5)** | **.002 (-1.4)** |
|  | **Brainstem** | **Bil** | **-.02 (10.2)** | **0.012** | **.002 (0.9)** | **.000 (0.0)** | **.001 (0.8)** | **.003 (1.2)** | **-.03 (26.7)** | **0.013** | **.001 (-0.4)** | **.043 (-15.8)** |
|  | **Caudate** | **Left** | **-.02 (5.7)** | **0.027** | **.015 (5.2)** | **.004 (1.7)** | **.000 (0.0)** | **.001 (0.6)** | **-.03 (20.9)** | **-0.003** | **.000 (-0.1)** | **.000 (-0.1)** |
|  |  | **Right** | **-.01 (1.6)** | **--** | **.011 (4.0)** | **.003 (1.4)** | **.000 (0.3)** | **.017 (6.0)** | **-.03 (37.5)** | **0.004** | **.000 (-0.4)** | **.004 (-1.8)** |
|  | **Cerebellum** | **Left** | **-.02 (3.8)** | **--** | **.002 (1.2)** | **.000 (0.1)** | **.002 (1.2)** | **.001 (0.4)** | **-.04 (44.2)** | **0.004** | **.000 (-0.4)** | **.005 (-2.2)** |
|  |  | **Right** | **-.02 (6.5)** | **0.019** | **.003 (1.5)** | **.001 (0.6)** | **.000 (0.3)** | **.000 (0.3)** | **-.04 (30.9)** | **0.006** | **.000 (0.2)** | **.005 (2.4)** |
|  | **Hippocampus** | **Left** | **-.06 (28.6)** | **0.030** | **.002 (1.1)** | **.001 (0.7)** | **.003 (-1.3)** | **.002 (-1.0)** | **-.06 (81.6)** | **-0.005** | **.001 (-0.6)** | **.000 (-0.1)** |
|  |  | **Right** | **-.06 (46.7)** | **0.019** | **.000 (-0.1)** | **.000 (-0.1)** | **.001 (-0.5)** | **.000 (-0.0)** | **-.05 (69.6)** | **-0.010** | **.000 (-0.2)** | **.002 (-1.2)** |
|  | **Pallidum** | **Left** | **-.02 (1.9)** | **--** | **.000 (0.3)** | **.001 (0.5)** | **.001 (0.7)** | **.002 (0.9)** | **-.01 (1.4)** | **--** | **.001 (0.8)** | **.045 (16.0)** |
|  |  | **Right** | **-.01 (1.7)** | **--** | **.003 (-1.5)** | **.003 (-1.4)** | **.000 (-0.2)** | **.004 (-1.6)** | **-.00 (0.2)** | **--** | **.000 (-0.2)** | **.017 (-6.4)** |
|  | **Putamen** | **Left** | **-.05 (22.6)** | **0.031** | **.005 (2.0)** | **.001 (0.6)** | **.003 (-1.3)** | **.009 (-3.6)** | **-.03 (23.1)** | **0.005** | **.000 (-0.3)** | **.006 (-2.7)** |
|  |  | **Right** | **-.04 (18.8)** | **0.020** | **.006 (2.6)** | **.002 (0.9)** | **.004 (-1.7)** | **.003 (-1.5)** | **-.04 (56.8)** | **0.003** | **.000 (-0.2)** | **.001 (-0.4)** |
|  | **Thalamus** | **Left** | **-.07 (25.2)** | **0.010** | **.000 (-0.1)** | **.000 (-0.0)** | **.003 (-1.3)** | **.009 (-3.5)** | **-.04 (40.2)** | **-0.006** | **.002 (-1.0)** | **.037 (-13.9)** |
|  |  | **Right** | **-.07 (32.8)** | **0.007** | **.000 (0.0)** | **.000 (0.1)** | **.003 (-1.3)** | **.004 (-1.8)** | **-.06 (69.0)** | **-0.011** | **.001 (-0.5)** | **.002 (-1.4)** |
|  | **CC anterior** | **Bil** | **-.01 (0.7)** | **--** | **.000 (-0.2)** | **.000 (-0.0)** | **.001 (0.8)** | **.000 (0.1)** | **-.02 (6.5)** | **0.000** | **.002 (-1.1)** | **.000 (-0.2)** |
|  | **CC central** | **Bil** | **-.04 (9.0)** | **0.005** | **.000 (0.2)** | **.000 (0.1)** | **.001 (-0.7)** | **.000 (-0.1)** | **-.02 (9.6)** | **-0.004** | **.000 (-0.1)** | **.001 (-0.8)** |
|  | **CC mid anterior** | **Bil** | **-.04 (8.6)** | **-0.001** | **.001 (0.6)** | **.000 (0.3)** | **.000 (-0.2)** | **.001 (-0.7)** | **-.03 (8.8)** | **-0.004** | **.001 (0.7)** | **.003 (1.5)** |
|  | **CC mid posterior** | **Bil** | **-.03 (7.8)** | **0.012** | **.000 (-0.1)** | **.000 (-0.0)** | **.000 (-0.3)** | **.000 (-0.0)** | **-.03 (16.0)** | **-0.007** | **.005 (-2.0)** | **.002 (-1.2)** |
|  | **CC posterior** | **Bil** | **-.01 (0.7)** | **--** | **.000 (0.3)** | **.000 (0.3)** | **.000 (0.4)** | **.001 (0.5)** | **-.01 (1.4)** | **--** | **.000 (-0.3)** | **.001 (-0.8)** |
|  | **Cerebellum WM** | **Left** | **-.02 (2.5)** | **--** | **.000 (0.2)** | **.001 (0.4)** | **.000 (0.0)** | **.004 (1.6)** | **-.02 (5.2)** | **-0.009** | **.000 (0.4)** | **.000 (0.1)** |
|  |  | **Right** | **-.03 (3.2)** | **--** | **.001 (-0.7)** | **.002 (-0.9)** | **.000 (0.2)** | **.008 (3.0)** | **-.03 (7.0)** | **-0.007** | **.000 (0.0)** | **.001 (0.6)** |
|  | **Cerebral WM** | **Left** | **-.03 (45.5)** | **0.001** | **.001 (-0.6)** | **.001 (-0.8)** | **.006 (2.5)** | **.002 (1.1)** | **-.04 (56.2)** | **0.001** | **.000 (-0.2)** | **.000 (-0.4)** |
|  |  | **Right** | **-.03 (44.2)** | **-0.005** | **.003 (-1.2)** | **.002 (-1.0)** | **.005 (2.3)** | **.001 (0.5)** | **-.03 (38.5)** | **-0.002** | **.001 (-0.6)** | **.000 (-0.1)** |
|  |  | **Bil** | **--** | **--** | **--** | **--** | **--** | **--** | **-.03 (53.1)** | **0.000** | **.001 (-0.4)** | **.000 (-0.1)** |
|  | **WM hypointensities** | **Bil** | **.06 (35.0)** | **-0.031** | **.021 (7.7)** | **.024 (8.8)** | **.000 (-0.1)** | **.008 (-3.3)** | **-.04 (56.0)** | **-0.009** | **.020 (7.9)** | **.001 (0.7)** |
|  | **Non WM hypointensities** | **Bil** | **.00 (0.3)** | **--** | **.002 (1.0)** | **.001 (0.7)** | **.000 (0.1)** | **.001 (0.6)** | **-.03 (3.6)** | **--** | **.000 (0.1)** | **.002 (1.0)** |
|  | **Brain Seg** | **Bil** | **-.03 (31.9)** | **0.036** | **.001 (0.4)** | **.001 (0.6)** | **.000 (0.0)** | **.005 (2.0)** | **-.04 (129.5)** | **0.001** | **.000 (-0.1)** | **.022 (-8.2)** |
|  | **Brain Seg not vent** | **Bil** | **-0.04 (49.2)** | **0.039** | **.000 (0.1)** | **.002 (1.0)** | **.000 (-0.2)** | **.006 (-2.6)** | **-.04 (160.2)** | **0.002** | **.001 (-0.7)** | **.024 (-9.3)** |
|  | **Brain Seg not vent surf** | **Bil** | **-.04 (58.3)** | **0.038** | **.000 (0.2)** | **.003 (1.5)** | **.000 (0.1)** | **.008 (3.2)** | **-.05 (161.3)** | **0.002** | **.001 (-0.7)** | **.023 (-9.1)** |
|  | **Cortex** | **Left** | **-.05 (21.0)** | **0.091** | **.000 (0.2)** | **.001 (0.6)** | **.003 (-1.5)** | **.008 (-3.2)** | **-.05 (100.6)** | **0.006** | **.000 (-0.2)** | **.045 (-16.3)** |
|  |  | **Right** | **-.04 (15.0)** | **0.074** | **.001 (0.6)** | **.003 (1.3)** | **.000 (-0.3)** | **.001 (-0.8)** | **-.05 (96.3)** | **0.005** | **.001 (-0.6)** | **.041 (-14.7)** |
|  |  | **Bil** | **--** | **--** | **--** | **--** | **--** | **--** | **-.05 (105.7)** | **0.005** | **.000 (-0.4)** | **.047 (-16.8)** |
|  | **eICV** | **Bil** | **-.00 (0.8)** | **--** | **.001 (0.5)** | **.000 (0.3)** | **.000 (0.3)** | **.008 (3.1)** | **-.00 (0.5)** | **--** | **.002 (1.3)** | **.000 (0.0)** |
|  | **Subcort gray** | **Bil** | **-.06 (59.7)** | **0.021** | **.003 (1.3)** | **.000 (0.3)** | **.003 (-1.5)** | **.003 (-1.5)** | **-.05 (113.5)** | **-0.005** | **.002 (-1.1)** | **.001 (-0.9)** |
|  | **Supratentorial** | **Bil** | **-.03 (42.4)** | **0.036** | **.001 (0.4)** | **.002 (1.1)** | **.000 (0.1)** | **.006 (2.3)** | **-.04 (113.2)** | **0.001** | **.000 (-0.1)** | **.018 (-7.0)** |
|  | **Supratentorial not vent** | **Bil** | **-.04 (66.9)** | **0.038** | **.000 (0.0)** | **.004 (1.9)** | **.000 (-0.1)** | **.008 (-3.2)** | **-.04 (146.2)** | **0.002** | **.001 (-0.7)** | **.022 (-8.4)** |
|  | **Total gray** | **Bil** | **-.05 (28.6)** | **0.073** | **.001 (0.8)** | **.002 (0.9)** | **.001 (-0.8)** | **.005 (-2.2)** | **-.05 (130.4)** | **0.004** | **.001 (-0.5)** | **.044 (-16.1)** |
|  | **Choroid plexus** | **Left** | **-.05 (24.4)** | **-0.002** | **.003 (1.2)** | **.001 (0.6)** | **.014 (5.0)** | **.011 (4.0)** | **-.03 (14.9)** | **-0.036** | **.001 (0.8)** | **.005 (2.5)** |
|  |  | **Right** | **-.04 (16.7)** | **0.002** | **.000 (0.1)** | **.000 (0.3)** | **.022 (7.3)** | **.014 (4.8)** | **-.03 (14.5)** | **-0.037** | **.001 (0.6)** | **.009 (4.0)** |
|  | **Optic chiasm** | **Bil** | **-.01 (0.6)** | **--** | **.000 (0.2)** | **.000 (0.1)** | **.005 (2.1)** | **.012 (4.4)** | **-.00 (0.1)** | **--** | **.000 (-0.2)** | **.000 (-0.1)** |
|  | **Ventral DC** | **Left** | **-.06 (30.2)** | **0.014** | **.000 (0.3)** | **.000 (0.2)** | **.000 (-0.3)** | **.003 (-1.3)** | **-.04 (9.0)** | **0.000** | **.002 (-1.3)** | **.001 (-0.9)** |
|  |  | **Right** | **-.06 (30.7)** | **0.010** | **.001 (-0.4)** | **.002 (-1.0)** | **.001 (-0.7)** | **.023 (-7.9)** | **-.05 (17.8)** | **-0.002** | **.000 (-0.3)** | **.000 (-0.1)** |
|  | **Ventricle choroid** | **Bil** | **-.06 (126.4)** | **-0.005** | **.032 (12.1)** | **.016 (6.3)** | **.014 (5.6)** | **.008 (3.4)** | **--** | **--** | **--** | **--** |
|  | **Vessel** | **Left** | **-.00 (0.2)** | **--** | **.000 (0.2)** | **.000 (0.2)** | **.001 (-0.6)** | **.001 (-0.5)** | **.00 (0.1)** | **--** | **.001 (0.4)** | **.002 (1.1)** |
|  |  | **Right** | **-.01 (1.9)** |  | **.000 (-0.2)** | **.000 (-0.2)** | **.000 (0.0)** | **.000 (0.0)** | **.00 (0.0)** | **--** | **.000 (-0.3)** | **.007 (-3.1)** |
| **Intensity** | **3rd Ventricle** | **Bil** | **-.05 (19.1)** | **0.009** | **.001 (0.8)** | **.001 (0.7)** | **.021 (-7.3)** | **.010 (-3.6)** | **-.03 (13.5)** | **-0.045** | **.000 (0.2)** | **.001 (0.5)** |
|  | **4th Ventricle** | **Bil** | **-.08 (23.6)** | **0.026** | **.000 (-0.3)** | **.000 (-0.3)** | **.007 (-2.7)** | **.001 (-0.5)** | **-.02 (2.0)** | **--** | **.000 (0.0)** | **.003 (1.8)** |
|  | **5th Ventricle** | **Bil** | **-.02 (0.5)** | **--** | **.001 (0.7)** | **.002 (1.1)** | **.000 (-0.3)** | **.002 (-1.1)** | **.02 (0.5)** | **--** | **.000 (0.1)** | **.005 (2.3)** |
|  | **Inf lat vent** | **Left** | **-.05 (14.4)** | **-0.008** | **.015 (-5.4)** | **.004 (-1.8)** | **.008 (-3.0)** | **.002 (-1.0)** | **-.05 (13.7)** | **-0.047** | **.000 (-0.1)** | **.008 (-3.5)** |
|  |  | **Right** | **-.04 (8.4)** | **-0.003** | **.002 (-1.0)** | **.001 (-0.4)** | **.002 (-0.8)** | **.000 (-0.1)** | **-.05 (11.3)** | **-0.044** | **.000 (0.0)** | **.004 (1.8)** |
|  | **Lat vent** | **Left** | **-.03 (4.5)** | **-0.007** | **.000 (0.3)** | **.001 (0.5)** | **.007 (-2.6)** | **.000 (-0.0)** | **-.03 (6.5)** | **-0.081** | **.000 (0.1)** | **.005 (2.4)** |
|  |  | **Right** | **-.03 (6.4)** | **-0.005** | **.000 (0.1)** | **.000 (0.2)** | **.007 (-2.8)** | **.000 (-0.1)** | **-.03 (9.1)** | **-0.077** | **.000 (0.2)** | **.006 (3.0)** |
|  | **CSF total** | **Bil** | **-.05 (11.1)** | **0.019** | **.000 (0.2)** | **.000 (0.3)** | **.009 (-3.5)** | **.003 (-1.4)** | **-.04 (14.1)** | **-0.058** | **.000 (0.0)** | **.010 (4.5)** |
|  | **Accumbens** | **Left** | **-.10 (22.6)** | **-0.047** | **.000 (0.1)** | **.000 (0.3)** | **.013 (4.5)** | **.038 (12.4)** | **.03 (3.0)** | **--** | **.000 (-0.1)** | **.032 (-11.8)** |
|  |  | **Right** | **-.10 (22.8)** | **-0.055** | **.000 (-0.1)** | **.000 (-0.0)** | **.007 (2.8)** | **.028 (9.5)** | **.04 (5.8)** | **-0.094** | **.000 (0.0)** | **.043 (16.2)** |
|  | **Amygdala** | **Left** | **-.03 (1.5)** | **--** | **.000 (-0.1)** | **.000 (-0.0)** | **.001 (-0.5)** | **.002 (-0.8)** | **.04 (3.6)** | **--** | **.000 (0.0)** | **.008 (3.4)** |
|  |  | **Right** | **-.03 (2.3)** | **--** | **.001 (-0.4)** | **.001 (-0.6)** | **.001 (0.6)** | **.006 (2.4)** | **.04 (4.1)** | **-0.099** | **.000 (0.0)** | **.008 (3.3)** |
|  | **Brainstem** | **Bil** | **-.07 (7.0)** | **0.058** | **.000 (-0.1)** | **.000 (-0.3)** | **.000 (0.0)** | **.001 (0.8)** | **-.03 (1.3)** | **--** | **.001 (-0.6)** | **.033 (-11.9)** |
|  | **Caudate** | **Left** | **-.05 (8.0)** | **-0.025** | **.000 (0.0)** | **.000 (0.2)** | **.002 (1.1)** | **.025 (8.4)** | **-.01 (0.7)** | **--** | **.000 (0.0)** | **.003 (1.7)** |
|  |  | **Right** | **-.05 (6.0)** | **-0.034** | **.000 (-0.2)** | **.000 (-0.1)** | **.004 (1.7)** | **.024 (8.1)** | **.00 (0.2)** | **--** | **.000 (-0.4)** | **.001 (-0.9)** |
|  | **Cerebellum** | **Left** | **-.12 (34.8)** | **0.041** | **.000 (0.0)** | **.002 (0.8)** | **.000 (0.0)** | **.010 (3.6)** | **.03 (4.5)** | **-0.062** | **.000 (0.2)** | **.003 (1.8)** |
|  |  | **Right** | **-.15 (47.9)** | **0.025** | **.001 (0.5)** | **.000 (0.1)** | **.001 (-0.7)** | **.000 (-0.2)** | **.00 (0.0)** | **--** | **.000 (0.2)** | **.003 (1.7)** |
|  | **Hippocampus** | **Left** | **-.03 (1.8)** | **--** | **.000 (-0.2)** | **.000 (-0.2)** | **.006 (-2.4)** | **.001 (-0.6)** | **.02 (2.1)** | **--** | **.001 (-0.6)** | **.014 (-5.5)** |
|  |  | **Right** | **-.07 (10.2)** | **0.013** | **.000 (-0.4)** | **.000 (-0.3)** | **.001 (-0.6)** | **.000 (-0.3)** | **.02 (1.0)** | **--** | **.000 (0.0)** | **.004 (1.8)** |
|  | **Pallidum** | **Left** | **-.00 (0.1)** | **--** | **.001 (0.4)** | **.000 (0.0)** | **.001 (-0.6)** | **.009 (-3.3)** | **-.01 (0.3)** | **--** | **.001 (0.8)** | **.011 (4.3)** |
|  |  | **Right** | **-.01 (0.4)** | **--** | **.002 (1.2)** | **.000 (0.4)** | **.000 (0.0)** | **.002 (1.1)** | **.05 (5.2)** | **-0.007** | **.001 (0.5)** | **.003 (1.7)** |
|  | **Putamen** | **Left** | **-.03 (2.3)** | **--** | **.001 (-0.5)** | **.000 (-0.1)** | **.004 (-1.6)** | **.001 (-0.6)** | **.01 (0.3)** | **--** | **.000 (0.1)** | **.010 (4.2)** |
|  |  | **Right** | **-.00 (0.2)** | **--** | **.000 (-0.1)** | **.000 (-0.0)** | **.000 (0.1)** | **.000 (0.3)** | **.04 (8.5)** | **-0.066** | **.000 (0.3)** | **.000 (0.3)** |
|  | **Thalamus** | **Left** | **-.06 (7.2)** | **0.027** | **.000 (-0.2)** | **.000 (-0.1)** | **.005 (-2.0)** | **.021 (-7.3)** | **-.01 (0.5)** | **--** | **.000 (0.0)** | **.016 (5.9)** |
|  |  | **Right** | **-.06 (8.0)** | **0.035** | **.000 (-0.1)** | **.000 (-0.1)** | **.007 (-2.8)** | **.017 (-5.8)** | **.01 (0.3)** | **--** | **.001 (-0.5)** | **.010 (-4.0)** |
|  | **CC anterior** | **Bil** | **-.04 (5.5)** | **0.017** | **.006 (-2.4)** | **.006 (-2.4)** | **.001 (0.4)** | **.004 (1.7)** | **-.03 (3.5)** | **--** | **.000 (0.3)** | **.015 (5.8)** |
|  | **CC central** | **Bil** | **-.04 (4.6)** | **0.024** | **.000 (-0.1)** | **.000 (-0.3)** | **.000 (-0.3)** | **.000 (-0.0)** | **-.03 (2.1)** | **--** | **.001 (0.5)** | **.025 (9.0)** |
|  | **CC mid anterior** | **Bil** | **-.03 (3.3)** | **--** | **.001 (-0.5)** | **.000 (-0.2)** | **.000 (0.0)** | **.001 (0.6)** | **-.04 (4.0)** | **0.090** | **.000 (-0.3)** | **.020 (-7.6)** |
|  | **CC mid posterior** | **Bil** | **-.06 (22.1)** | **0.025** | **.001 (0.7)** | **.000 (0.3)** | **.006 (-2.5)** | **.009 (-3.3)** | **-.03 (3.8)** | **--** | **.002 (1.4)** | **.031 (11.5)** |
|  | **CC posterior** | **Bil** | **-.04 (8.2)** | **0.031** | **.000 (0.1)** | **.000 (0.2)** | **.000 (0.3)** | **.000 (0.0)** | **-.02 (0.9)** | **--** | **.001 (-0.5)** | **.000 (-0.1)** |
|  | **Cerebellum WM** | **Left** | **-.11 (12.8)** | **0.097** | **.000 (-0.1)** | **.000 (-0.2)** | **.008 (-3.2)** | **.009 (-3.3)** | **-.01 (0.3)** | **--** | **.000 (0.1)** | **.000 (0.3)** |
|  |  | **Right** | **-.10 (12.2)** | **0.062** | **.000 (-0.2)** | **.000 (-0.1)** | **.013 (-4.7)** | **.003 (-1.4)** | **-.01 (0.3)** | **--** | **.001 (-0.5)** | **.000 (-0.4)** |
|  | **WM hypointensities** | **Bil** | **-.02 (1.7)** | **--** | **.000 (0.1)** | **.001 (0.5)** | **.001 (-0.7)** | **.022 (-7.5)** | **-.06 (23.2)** | **0.000** | **.001 (-0.3)** | **.001 (-0.4)** |
|  | **Non WM hypointensities** | **Bil** | **-.00 (0.0)** | **--** | **.000 (-0.1)** | **.000 (-0.0)** | **.000 (0.4)** | **.003 (1.2)** | **.01 (0.3)** | **--** | **.000 (0.1)** | **.005 (2.3)** |
|  | **Choroid plexus** | **Left** | **-.06 (25.1)** | **0.011** | **.000 (-0.1)** | **.000 (-0.0)** | **.018 (-6.4)** | **.012 (-4.4)** | **-.05 (32.9)** | **-0.003** | **.001 (-0.5)** | **.001 (-0.6)** |
|  |  | **Right** | **-.05 (15.3)** | **-0.009** | **.000 (0.0)** | **.001 (0.6)** | **.032 (-10.7)** | **.014 (-5.0)** | **-.06 (37.9)** | **-0.009** | **.001 (-0.8)** | **.005 (-2.2)** |
|  | **Optic chiasm** | **Bil** | **-.01 (0.5)** | **--** | **.000 (-0.1)** | **.003 (-1.4)** | **.000 (-0.1)** | **.000 (-0.4)** | **-.01 (0.3)** | **--** | **.000 (-0.4)** | **.004 (-1.9)** |
|  | **Ventral DC** | **Left** | **-.02 (0.9)** | **--** | **.001 (0.6)** | **.000 (0.2)** | **.000 (-0.4)** | **.001 (-0.4)** | **-.01 (0.2)** | **--** | **.000 (-0.1)** | **.028 (-10.1)** |
|  |  | **Right** | **-.02 (0.6)** | **--** | **.001 (0.4)** | **.000 (0.0)** | **.000 (0.0)** | **.003 (1.3)** | **.00 (0.0)** | **--** | **.000 (-0.1)** | **.018 (-6.8)** |
|  | **Vessel** | **Left** | **-.01 (0.4)** | **--** | **.000 (0.1)** | **.001 (0.5)** | **.004 (-1.9)** | **.000 (-0.1)** | **-.01 (0.5)** | **--** | **.000 (-0.3)** | **.006 (-2.6)** |
|  |  | **Right** | **-.02 (0.7)** | **--** | **.000 (0.0)** | **.000 (0.1)** | **.002 (-0.8)** | **.001 (-0.5)** | **-.02 (2.8)** | **--** | **.000 (0.1)** | **.017 (6.4)** |
