# Supplementary material for: Individual variations in ‘brain age’ relate to early-life factors more than to longitudinal brain change
Source: eLife. 2021 Nov 10;10:e69995. doi: 10.7554/eLife.69995 (PMC8580481; doi:10.7554/eLife.69995)
Supplement: Supplementary file 5. — Contact information and ethical comittees for the different cohorts. [file elife-69995-supp5.docx]

| **Lifebrain Consortium (**[**http://www.lifebrain.uio.no/about/**](http://www.lifebrain.uio.no/about/)**)** | | | |  |
| --- | --- | --- | --- | --- |
| **LCBC** | [http://www.oslobrains.no](http://www.oslobrains.no/) | Kristine B. Walhovd | [k.b.walhovd@psykologi.uio.no](mailto:k.b.walhovd@psykologi.uio.no) | Norwegian Regional Committee for Medical and Health Research Ethic; Regional Ethical Committee of South Norway |
| **BETULA** | <http://www.ufbi.umu.se/english> | Lars Nyberg | [lars.nyberg@umu.se](mailto:lars.nyberg@umu.se) | Regional Ethical Vetting Board at Umeå University |
| **BASE-II** | <https://www.mpib-berlin.mpg.de/en/research/lifespan-psychology> | Ulman Lindenberger | [lindenberger@mpib-berlin.mpg.de](mailto:lindenberger@mpib-berlin.mpg.de) | Ethics committee of the Charité-Universitätsmedizin *Berlin* |
| **Cam-CAN** | <https://www.cam-can.org/> | Lorraine K. Tyler & Richard Henson | [lktyler@csl.psychol.cam.ac.uk](mailto:lktyler@csl.psychol.cam.ac.uk) & [rik.henson@mrc-cbu.cam.ac.uk](mailto:rik.henson@mrc-cbu.cam.ac.uk) | Cambridgeshire 2 Research Ethics Committee |
| **UB** | <http://www.ub.edu/bbslab/bbslab/> | David Bartrés-Faz | [dbartres@ub.edu](mailto:dbartres@ub.edu) | Comisión de Bioética de la Universidad de Barcelona and Hospital Clinic |
| **AIBL*** | <https://aibl.csiro.au/research/> | Christopher Rowe | [christopher.rowe@austin.org.au](mailto:christopher.rowe@austin.org.au) | Institutional ethics committees of Austin Health, StVincent’s Health, Hollywood Private Hospital and Edith Cowan University |
