# Supplementary material for: Individual variations in ‘brain age’ relate to early-life factors more than to longitudinal brain change
Source: eLife. 2021 Nov 10;10:e69995. doi: 10.7554/eLife.69995 (PMC8580481; doi:10.7554/eLife.69995)
Supplement: Supplementary file 6. — Data acquisition parameters for the T1w sequences. *UK Biobank employed three scanners of the same model and with equivalent parameters (Cheadle, Reading, and Newcastle centers). **AIBL does not belong to the Lifebrain consortium but was included in the Lifebrain replication dataset. [file elife-69995-supp6.docx]

| **Sample** | **Scanner** | **Sequence** | **Tesla** | **Slices** | **Voxel size (mm)** | **Time parameters**  **(TR / TE / TI [ms])** | **Other parameters**  **(FA / FOV [∘/mm])** |
| --- | --- | --- | --- | --- | --- | --- | --- |
| **UK Biobank (main sample)** | | | | | | | |
| UK Biobank* | Skyra Siemens | 3D MP-RAGE | 3.0 | 256 | 1 x 1 x 1 | 2,000/-/880 |  |
| **Lifebrain (replication sample)** | | | | | | | |
| LCBC | Avanto Siemens | 3D MP-RAGE | 1.5 | 160 | 1.25 x 1.25 x 1.25 | 2,400/3.61/1,000 | 8/240x240 |
|  | Skyra Siemens | 3D MP-RAGE | 3.0 | 176 | 1 x 1 x 1 | 2,300/2.98/850 | 8/256 x 256 |
|  | Prisma Siemens | 3D MP-RAGE | 3.0 | 208 | 1 x 1 x 1 | 2,400/2.22/1,000 | 8/240 x 256 |
| Cam-CAN | Tim Trio Siemens | 3D MP-RAGE | 3.0 | 192 | 1 x 1 x 1 | 2,250/2.98/900 | 9/256 x 240 |
| Base-II | Tim Trio Siemens | 3D MP-RAGE | 3.0 | 176 | 1 x 1 x 1 | 2,500/4.77/1,100 | 7/256 x 256 |
| Betula | Discovery GE | 3D FSPGR | 3.0 | 176 | 1 x 1 x 1 | 8.19/3.2/450 | 12/250 x 250 |
| UB | Tim Trio Siemens | 3D MP-RAGE | 3.0 | 240 | 1 x 1 x 1 | 2,300/2.98/900 | 9/256 x 256 |
| AIBL** | Avanto Siemens | 3D MP-RAGE | 1.5 | 160 | 1 x 1 x 1.2 | 2,300/2.98/900 | 9/240 x 256 |
|  | Verio Siemens | 3D MP-RAGE | 3.0 | 160 | 1 x 1 x 1.2 | 2,300/2.98/900 | 9/240 x 256 |
|  | Tim Trio Siemens | 3D MP-RAGE | 3.0 | 160 | 1 x 1 x 1.2 | 2,300/2.98/900 | 9/240 x 256 |
