# Supplementary material for: Individual variations in ‘brain age’ relate to early-life factors more than to longitudinal brain change
Source: eLife. 2021 Nov 10;10:e69995. doi: 10.7554/eLife.69995 (PMC8580481; doi:10.7554/eLife.69995)
Supplement: Transparent reporting form [file elife-69995-transrepform1.pdf]

Westbrook Centre
Milton Road
Cambridge CB4 1YG, UK

P 01223 855340
W elifesciences.org
T @elife

eLife's transparent reporting form

We encourage authors to provide detailed information *within their submission* to facilitate the interpretation and replication of experiments. Authors can upload supporting documentation to indicate the use of appropriate reporting guidelines for health-related research (see EQUATOR Network), life science research (see the BioSharing Information Resource), or the ARRIVE guidelines for reporting work involving animal research. Where applicable, authors should refer to any relevant reporting standards documents in this form.

If you have any questions, please consult our Journal Policies and/or contact us: editorial@elifesciences.org.

Sample-size estimation

- You should state whether an appropriate sample size was computed when the study was being designed
- You should state the statistical method of sample size computation and any required assumptions
- If no explicit power analysis was used, you should describe how you decided what sample (replicate) size (number) to use

Please outline where this information can be found within the submission (e.g., sections or figure legends), or explain why this information doesn't apply to your submission:

For all analyses sample size was determined based on data availability. No formal analyses were performed to predetermine sample sizes. We gathered as much data as we could, from both cross-sectional and longitudinal observations. With the available data both for the main and the replication datasets, one should be able to detect effects explaining around .2-.3% of the variance as significant according to a frequentist threshold of $p < .05$. This information is detailed in the **Methods section**.

Replicates

- You should report how often each experiment was performed
- You should include a definition of biological versus technical replication
- The data obtained should be provided and sufficient information should be provided to indicate the number of independent biological and/or technical replicates
- If you encountered any outliers, you should describe how these were handled
- Criteria for exclusion/inclusion of data should be clearly stated
- High-throughput sequence data should be uploaded before submission, with a private link for reviewers provided (these are available from both GEO and ArrayExpress)

Please outline where this information can be found within the submission (e.g., sections or figure legends), or explain why this information doesn't apply to your submission:

eLife Sciences Publications, Ltd is a limited liability non-profit non-stock corporation incorporated in the State of Delaware, USA, with company number 5030732, and is registered in the UK with company number FC030576 and branch number BR015634 at the address Westbrook Centre, Milton Road, Cambridge CB4 1YG.

We replicated the main findings using 1) a different algorithm for computing brain age (technical replicate) and 2) an independent sample (biological replicate). Additional control analyses were performed controlling for additional variables (interval between observations and removing bias correction steps during the preprocessing. The Replication for the observed relationship between PGS scores/Birth weight and brain age was only possible across algorithm as the independent replication sample did not have genetic nor birth weight available information (**see Introduction and Methods section)**.

We used already available datasets. While most inclusion/exclusion criteria were common across datasets, the specific details varied slightly across cohorts. The manuscript provides key references where specific inclusion/exclustion criteria is outlined for each cohort (**see Methods/Participants and Samples section**). In addition to the existing criteria, we additionally removed individual observations where individuals showed evidence of cognitive impairment, extreme outliers attributed to preprocessing errors, and observations of individuals younger than 18 years.
In technical replication analyses we removed datapoints considered as extreme outliers (>6SD of the mean) (**see Methods/Lifebrain-specific steps section**).

eLife Sciences Publications, Ltd is a limited liability non-profit non-stock corporation incorporated in the State of Delaware, USA, with company number 5030732, and is registered in the UK with company number FC030576 and branch number BR015634 at the address Westbrook Centre, Milton Road, Cambridge CB4 1YG.

Statistical reporting

- Statistical analysis methods should be described and justified
- Raw data should be presented in figures whenever informative to do so (typically when N per group is less than 10)
- For each experiment, you should identify the statistical tests used, exact values of N, definitions of center, methods of multiple test correction, and dispersion and precision measures (e.g., mean, median, SD, SEM, confidence intervals; and, for the major substantive results, a measure of effect size (e.g., Pearson's r, Cohen's d)
- Report exact p-values wherever possible alongside the summary statistics and 95% confidence intervals. These should be reported for all key questions and not only when the p-value is less than 0.05.

Please outline where this information can be found within the submission (e.g., sections or figure legends), or explain why this information doesn't apply to your submission:

eLife Sciences Publications, Ltd is a limited liability non-profit non-stock corporation incorporated in the State of Delaware, USA, with company number 5030732, and is registered in the UK with company number FC030576 and branch number BR015634 at the address Westbrook Centre, Milton Road, Cambridge CB4 1YG.

All main figures (**Fig.1-3**) show raw data.

Brain age prediction: We used gradient boosting algorithm (as implemented in R's XGBOOST). The model was optimized in the training set using a 10-fold cross-validation randomized hyper-parameters search and then brain age was predicted on the test dataset. For replication across algorithms we used an available routine based on LASSO as implemented in glmnet R-package. See https://james-cole.github.io/UKBiobank-Brain-Age/ for more details. Brain age delta scores were corrected for age-bias. See **Methods/Statistical analysis/Brain age prediction section for full description**.

We implemented either linear regression models or linear mixed models for most analyses. Relationship between cross-sectional brain age delta and brain age delta-long: Linear models controlling for age, sex, site, and estimated intracranial volume (eICV). In the Lifebrain replication dataset site was included as a random intercept and thus we employed linear mixed models. For post-hoc equivalence tests we re-did the same analyses but with a variable right-hand contrast (i.e. varying null hypothesis). Relationship between cross-sectional brain age delta and PCA change: The first PCA of change was obtained introducing all features with significant tp2-tp1 effects (one-sample t-test against 0, Bonferroni-corrected $p < 0.05$). The association was assessed using linear models controlling for age, sex, site, and estimated intracranial volume (eICV). The same models were used to assess the relationship between cross-sectional/longitudinal brain age delta with feature change in each individual feature (Bonferroni-corrected $p < 0.05$). Relationship between cross/long brain age and birth weight/PGS-BA: Linear mixed models were used to fit time (from baseline; years), birth weight/PGS-BA, and its interaction on brain age delta, using age at baseline, sex, scanner, and eICV as covariates. For PGS-BA we additionally included 10 covariates accounting for population structure.

Effects of interest: Relationship between cross-sectional brain age delta and brain age delta-long: Main effect of cross-sectional brain age delta. Relationship between cross-sectional/longitudinal brain age delta and PCA/feature change: Main effect of cross-sectional/longitudinal brain age delta. Relationship between cross/long brain age and birth weight/PGS-BA: Main effect of birth-weight/PGS-BA as well as its interaction with time.

All p-values were derived from t-scores. For linear mixed models, significance was assessed as implemented in lmerTest r-package. This package provides p-values for lmer model fits via Satterthwaite's degrees of freedom method. For linear regressions significance was assessed as provided by the "lm" function (stats R-package). **Methods/Statistical analysis/Higher-level analysis section for full description and Results section for an outline**.

All main analyses report N, estimates, confidence interval, p-values, and measures of effect size. Bonferroni-type correction for multiple comparisons are used when necessary. Exact p-values are reported if $p > 0.001$; else we provide t-values and effect size measures. See **Results section and Caption to figures**.

(For large datasets, or papers with a very large number of statistical tests, you may upload a single table file with tests, Ns, etc., with reference to sections in the manuscript.)

eLife Sciences Publications, Ltd is a limited liability non-profit non-stock corporation incorporated in the State of Delaware, USA, with company number 5030732, and is registered in the UK with company number FC030576 and branch number BR015634 at the address Westbrook Centre, Milton Road, Cambridge CB4 1YG.

Group allocation

- Indicate how samples were allocated into experimental groups (in the case of clinical studies, please specify allocation to treatment method); if randomization was used, please also state if restricted randomization was applied
- Indicate if masking was used during group allocation, data collection and/or data analysis

Please outline where this information can be found within the submission (e.g., sections or figure legends), or explain why this information doesn't apply to your submission:

> Randomization was not performed as individuals were not allocated into experimental groups.

Additional data files ("source data")

- We encourage you to upload relevant additional data files, such as numerical data that are represented as a graph in a figure, or as a summary table
- Where provided, these should be in the most useful format, and they can be uploaded as "Source data" files linked to a main figure or table
- Include model definition files including the full list of parameters used
- Include code used for data analysis (e.g., R, MatLab)
- Avoid stating that data files are "available upon request"

Please indicate the figures or tables for which source data files have been provided:

> **Supplementary Tables 1,2,4** include source data for **Fig 1c**, **Supplementary Fig. 3,4.**
> All non-default model parameters are described in the **Methods section.**
> **Code** for model generation and higher-level analysis is now attached to the submission as a *zip file.
> Source data necessary to reproduce the main text Figures (**Fig. 1-3**) is attached to the submission of as an *Rda file.

eLife Sciences Publications, Ltd is a limited liability non-profit non-stock corporation incorporated in the State of Delaware, USA, with company number 5030732, and is registered in the UK with company number FC030576 and branch number BR015634 at the address Westbrook Centre, Milton Road, Cambridge CB4 1YG.
